# Endothelial‐Derived CCL7 Promotes Macrophage Polarization and Aggravates Septic Acute Lung Injury via CCR1‐Mediated STAT1 Succinylation

**DOI:** 10.1002/advs.202506209

**Published:** 2025-08-04

**Authors:** Xue Li, Yuqin Long, Yunxi Zhu, Jiahui Gu, Ping Zhou, Changhong Miao

**Affiliations:** ^1^ Department of Anesthesiology Zhongshan Hospital Fudan University Shanghai China; ^2^ Shanghai Key Laboratory of Perioperative Stress and Protection Shanghai China

**Keywords:** CCL7, glycolysis, macrophage, sepsis, succinylation

## Abstract

Acute lung injury (ALI) is a significant complication of sepsis, wherein the interaction between pulmonary vascular endothelial cells and immune cells plays a pivotal role in the pathogenesis. In this study, it is demonstrated that secretion of chemokine C‐C motif ligand 7 (CCL7) by endothelial cells (ECs) induces metabolic reprogramming and M1 polarization of C‐C motif chemokine receptor 1‐positive (CCR1⁺) macrophages. It is noteworthy that mice with specific inhibition of endothelial‐derived CCL7 exhibit reduced severity of septic ALI, underscoring the critical role of CCL7 in the progression of sepsis. Mechanistically, activation of the CCL7–CCR1 axis enhances STAT1 succinylation through upregulation of KAT2A expression, leading to increased STAT1 binding to the promoter of glycolytic genes in macrophages. This epigenetic regulation modulates metabolic reprogramming and M1 polarization of macrophages, thereby driving inflammatory cascades in septic ALI. Furthermore, in sepsis models, Ccr1‐knockout (Ccr1^‐KO^) mice demonstrate attenuated lung inflammation and decreased mortality, highlighting the therapeutic potential of targeting the CCL7–CCR1 axis for the treatment of septic ALI. Collectively, findings provide novel insights into the metabolic reprogramming of macrophages and identify the CCL7–CCR1 axis as a promising therapeutic target for septic ALI.

## Graphical abstract

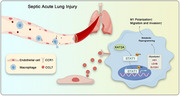

## 1 Introduction

Sepsis is a prevalent and critical syndrome associated with a high mortality rate in patients with infectious diseases, and effective treatment strategies remain limited.^[^ ^1^, ^2^ ^]^ The lung is the first and most commonly affected organ during sepsis.^[^ ^3^ ^]^ Septic acute lung injury (ALI) has a high mortality rate, and the mechanisms underlying its occurrence have not yet been fully elucidated. Lung macrophages, comprising resident alveolar macrophages and bone marrow‐derived recruited macrophages, serve as the predominant producers of inflammatory mediators.^[^ ^4^ ^]^ The infiltration of macrophages into lung tissue, along with associated inflammatory responses, is a critical factor in the development of septic ALI. These processes are primarily regulated by chemokines secreted by lung epithelial cells and endothelial cells.^[^ ^5^ ^]^ Therefore, investigating the chemokine‐mediated infiltration of macrophages is essential for understanding the progression of septic ALI.

Pulmonary vascular endothelial cells constitute ≈50% of the total number of lung cells and play a crucial role in the immune regulation of sepsis. Under sepsis conditions, endothelial cells secrete various chemokines, including C‐X‐C chemokine ligand 5 (CXCL5), chemokine C‐C motif ligand 2 (CCL2), and chemokine C‐C motif ligand 7 (CCL7).^[^ ^6^, ^7^ ^]^ Studies have demonstrated that endothelial‐derived CCL7 contributes to the regulation of macrophage infiltration and immune response.^[^ ^8^ ^]^ CCL7 triggers activation of cell surface chemokine receptors, with a particular emphasis on C‐C motif chemokine receptor 1 (CCR1). During sepsis, macrophages transition to a pro‐inflammatory phenotype characterized by increased glycolysis and inflammatory signaling.^[^ ^9^ ^]^ CCL7 influences the metabolic reprogramming and polarization of macrophages via CCR1 activation.^[^ ^10^, ^11^ ^]^ Consequently, targeting the CCL7–CCR1 axis in endothelial cell–macrophage interaction represents a promising therapeutic strategy for septic ALI.

Signal transducer and activator of transcription 1 (STAT1) is a central protein that plays a crucial role in the metabolic process of macrophages and participates in the regulation of macrophage polarization.^[^ ^12^, ^13^ ^]^ Various posttranslational modifications (PTMs), including phosphorylation, methylation, acetylation, and succinylation, modulate changes in STAT1 function.^[^ ^14^, ^15^, ^16^ ^]^ Succinylation refers to the enzymatic addition of a succinyl group to specific amino acid residues on proteins.^[^ ^17^ ^]^ Previous studies have indicated that succinylation plays a vital role in metabolic reprogramming in macrophages, although the precise mechanisms involved remain unclear.^[^ ^18^, ^19^ ^]^ Lysine acetyltransferase 2A (KAT2A) has recently been identified as a key enzyme that catalyzes succinylation.^[^ ^20^ ^]^ Research has shown that KAT2A regulates macrophage M1 polarization and contributes to sepsis‐related kidney and myocardial injury.^[^ ^21^, ^22^, ^23^ ^]^ However, the exact role of KAT2A in increasing the succinylation of its target proteins in septic ALI requires further clarification.

In this study, we demonstrated that during sepsis, pulmonary vascular endothelial cells secrete chemokine CCL7, which serves as a critical factor in promoting the infiltration of CCR1‐positive (CCR1^+^) macrophages into lung tissue. Activation of the CCL7–CCR1 axis enhances the succinylation of STAT1 by KAT2A, thereby driving metabolic reprogramming and promoting M1 polarization of macrophages. These findings provide novel insights into the interaction between pulmonary vascular endothelial cells and macrophages in the development of acute lung injury during sepsis.

## 2 Results

### 2.1 Inhibition of Endothelial‐Derived CCL7 Improves Septic ALI

Sepsis‐induced pulmonary vascular endothelial cell injury is a critical factor in the development of septic ALI.^[^ ^24^, ^25^ ^]^ To elucidate this process, we conducted an in‐depth analysis of single‐cell sequencing data (GSE207651) from the sham and cecal ligation and puncture (CLP) mouse models retrieved from the GEO database. A total of 11,826 and 8,074 cells were included in the sham and CLP groups, respectively (Figure S1A, Supporting Information). Based on marker gene expression, we classified these cells into 15 distinct clusters (Figure S1B, C, Supporting Information) and extracted the pan‐vascular endothelial cell cluster for further investigation. Gene Set Enrichment Analysis (GSEA) of differentially expressed genes (DEGs) between two groups revealed the activation of chemotaxis, inflammatory response, leukocyte cell‐cell adhesion, and positive regulation of programmed cell death pathways in pan‐vascular endothelial cells of septic mice (Figure S1D, E, Supporting Information). Subsequently, we established the sham and CLP mouse models, and the TUNEL staining confirmed that endothelial cells (ECs) in septic mice exhibited significant apoptosis‐related injury (Figure S1F, Supporting Information). These findings indicate that ECs may play a pivotal role in the inflammatory response and immune regulation in septic ALI.

**Figure 1:**
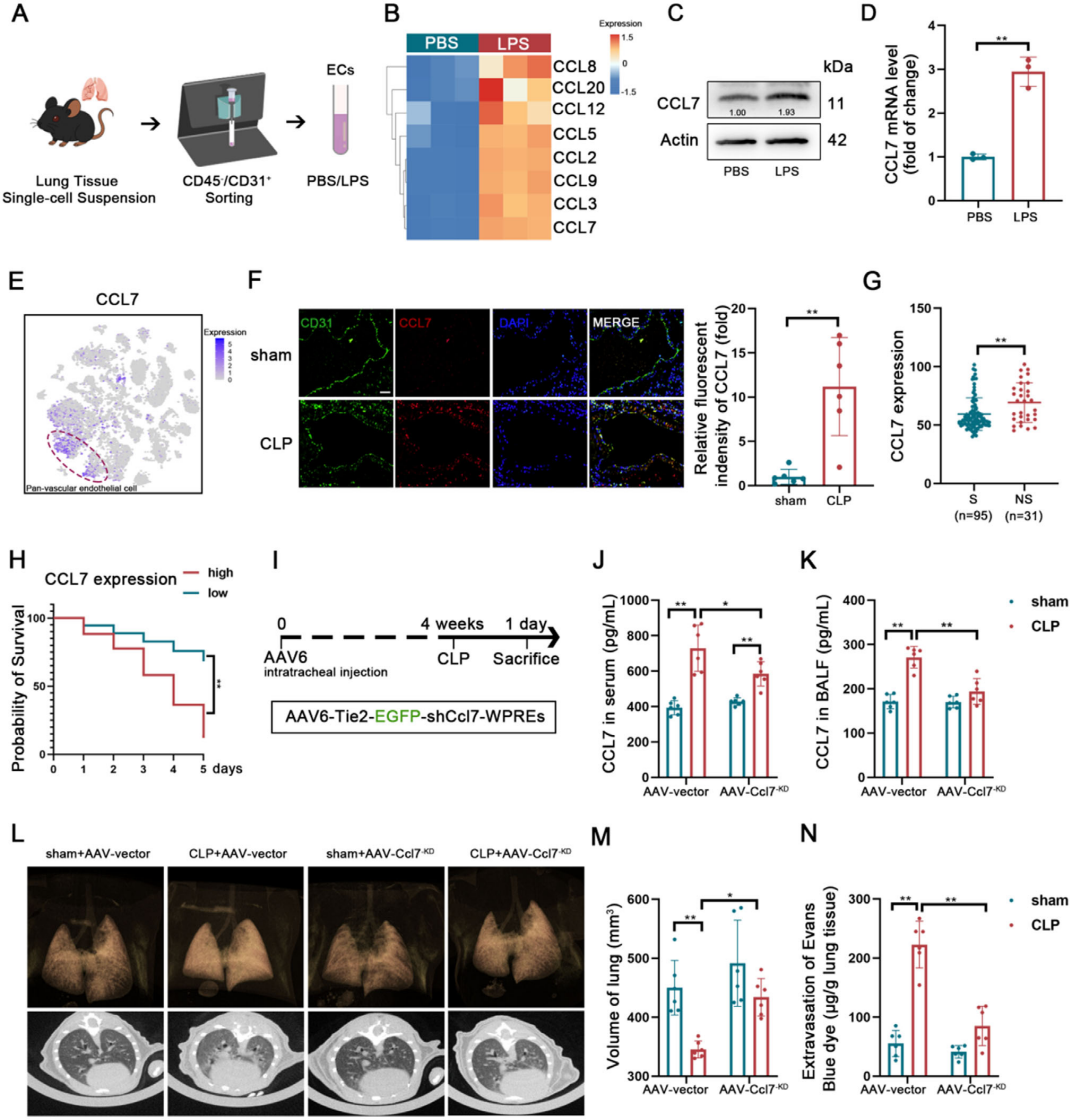
Inhibition of endothelial‐derived CCL7 improves septic ALI. A) Schematic diagram of the magnetic bead‐based sorting process for isolating ECs from the lung tissue of C57BL/6 mice. B) Heatmap illustrating the expression levels of classical chemokines derived from RNA sequencing data of ECs treated with or without LPS (10 µg mL^−1^) for 24 h (n = 3). C) Western blot analysis of CCL7 protein expression in ECs treated with or without LPS for 24 h. D) Relative mRNA expression of CCL7 in ECs treated with or without LPS for 24 h (n = 3). E) Dot plot showing the expression of CCL7 across all subclusters, visualized on the t‐SNE dimensionality reduction plot. F) Representative immunofluorescence co‐staining of CD31 (green) and CCL7 (red) in lung sections from the sham and CLP mice (scale bars: 50 µm). G) Analysis of CCL7 expression in whole‐blood RNA sequencing data from sepsis survivors (S, n = 95) and non‐survivors (NS, n = 31) in the GSE54514 database. H) Kaplan‐Meier survival curve comparing patients with high CCL7 expression versus low CCL7 expression in the GSE54514 database. I) Schematic diagram outlining the experimental design used to evaluate the efficacy of intratracheal delivery of AAV6‐Tie2‐EGFP‐shCcl7 in a mouse sepsis model. J, K) Serum (J) and BALF (K) CCL7 concentrations in AAV‐vector and AAV‐Ccl7^‐KD^ mice with or without sepsis (n = 6). L) 3D imaging and CT scans of lungs from AAV‐vector and AAV‐Ccl7^‐KD^ mice with or without sepsis. M) Quantification of micro‐CT‐derived non‐aerated lung volume as an indicator of lung consolidation (n = 6). N) Assessment of lung transvascular permeability by measuring Evans blue dye leakage in micrograms per gram of lung tissue in AAV‐vector and AAV‐Ccl7^‐KD^ mice with or without sepsis (n = 6). Data are presented as mean ± SD, ^*^ *p* < 0.05, ^**^ *p* < 0.01. Data in D, F, and G were analyzed by two‐tailed Student's *t*‐test. Data in H were analyzed by the Log‐rank test. Data in J, K, M, and N were analyzed by two‐way ANOVA with Tukey's post hoc test.

Next, we employed magnetic bead sorting to isolate CD45^‐^CD31⁺ ECs for further experiments (**Figures** **1**A, S2A, Supporting Information). Gene Ontology (GO) enrichment analysis of RNA sequencing data from lipopolysaccharide (LPS)‐treated ECs revealed increased activity of several cellular chemokine pathways (Figure S2B, Supporting Information). Among classical chemokines, LPS markedly increased **CCL7** expression in ECs (Figure 1B–D). Single‐cell sequencing data further confirmed that **CCL7** was predominantly expressed in pan‐vascular endothelial cell clusters (Figure 1E), and pseudo‐time analysis demonstrated an increasing trend of CCL7 expression in mouse pan‐vascular endothelial cells during sepsis (Figure S2C, Supporting Information). Immunofluorescence staining showed that CCL7 expression in the lung tissue of septic mice was upregulated and co‐localized with CD31⁺ ECs (Figure 1F). Additionally, the ELISA analyses of the serum and bronchoalveolar lavage fluid (BALF) from septic mice revealed elevated levels of CCL7 (Figure S2D, E, Supporting Information). Furthermore, CCL7 levels were also increased in the serum of the septic patients (Figure S2F, Supporting Information). To validate the clinical relevance of CCL7 in sepsis, we analyzed the GSE54514 RNA sequencing data from the peripheral blood of septic patients. The results indicated that **CCL7** levels were significantly higher in non‐survivors (NS) compared to survivors (S) (Figure 1G) and that CCL7 levels were positively correlated with APACHE II scores (Figure S2G, Supporting Information). Receiver operating characteristic (ROC) curve analysis (AUC = 0.67, Figure S2H, Supporting Information) suggested that CCL7 could serve as a prognostic marker. Kaplan‐Meier survival analysis revealed that patients with lower **CCL7** expression had improved survival outcomes compared to those with higher CCL7 expression (Figure 1H). Collectively, these findings suggest that **CCL7** expression increases during sepsis and influences patient outcomes, warranting further investigation into its precise role in septic ALI.

To explore the function of CCL7, we utilized adeno‐associated virus (AAV)‐type 6 carrying the **Tie2** promoter to specifically target ECs and inhibit **CCL7** expression via intratracheal injection (AAV‐Ccl7^‐KD^ mice, Figure 1I, Figure S2I, Supporting Information). This intervention partially reduced CCL7 levels in the serum and significantly suppressed **CCL7** expression in the BALF of septic mice (Figure 1J,K). Specific inhibition of endothelial‐derived CCL7 significantly decreased the mortality rate of septic mice (Figure S2J, Supporting Information). Chest CT imaging demonstrated that EC‐specific **CCL7** knockdown alleviated lung inflammation in septic mice (Figure 1L) and partially restored lung volume deficits caused by sepsis (Figure 1M). The histopathological analysis further confirmed that knocking down CCL7 reduced sepsis‐related lung injury scores (Figure S2K, L, Supporting Information), mitigated EC apoptosis (Figure S2M, Supporting Information), and alleviated sepsis‐induced pulmonary vascular hyperpermeability (Figure 1N).

### 2.2 CCL7 Regulates Infiltration and Inflammation of CCR1^+^ Macrophages

**Figure 2:**
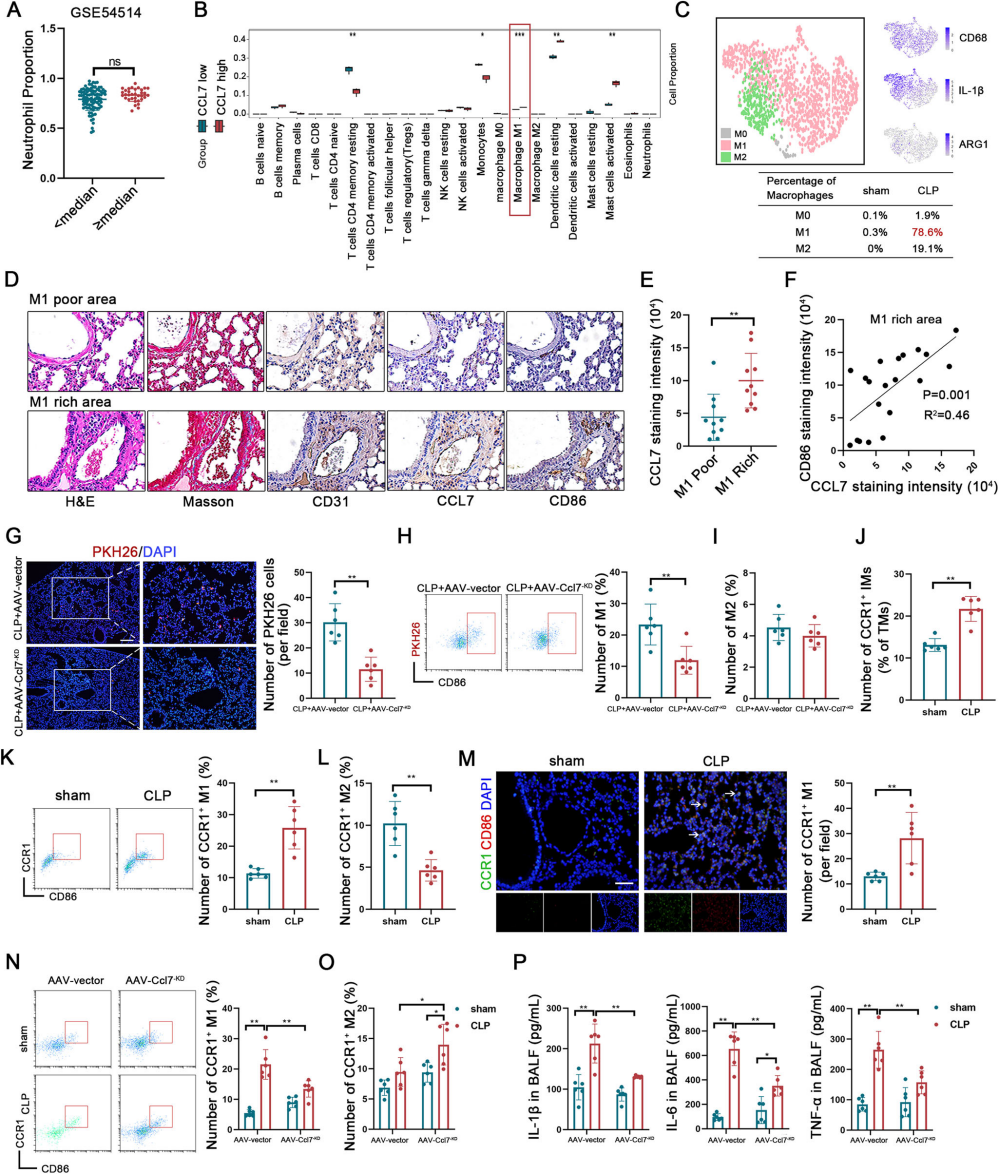
CCL7 regulates infiltration and inflammation of CCR1^+^ macrophages. A) Comparison of the proportions of neutrophil infiltration in patients with sepsis based on median CCL7 expression levels in the GSE54514 database. B) Immune cell proportions deconvoluted by CIBERSORT analysis for the CCL7‐low and CCL7‐high groups derived from RNA sequencing data of ECs (n = 3). C) t‐SNE plot illustrating the transcriptional identity of macrophages analyzed by single‐cell sequencing data. Annotations of cell clusters were shown (upper panel). The percentages of each macrophage subtype in the sham and CLP mice were presented (lower panel). D) Histopathological characterization using H&E and Masson staining along with immunohistochemical quantification of CD86^+^ macrophages (M1) and endothelial CCL7 expression (scale bars: 50 µm). E) Paired analysis of CCL7 expression in M1‐poor and M1‐rich areas (n = 10). F) Pearson correlation matrix assessing the relationship between CCL7 expression and CD86 expression (n = 20). G) Fluorescence imaging of lung tissue sections from AAV‐vector and AAV‐Ccl7^‐KD^ septic mice transfused with PKH26‐labeled BMDMs. The number of PKH26‐labeled BMDMs per field was quantified (scale bars: 200 µm, n = 6). H, I) Proportions of the CD86^+^ M1 macrophages (H) or CD206^+^ M2 (I) macrophages among the PKH26‐labeled BMDMs in the lung tissue of AAV‐vector and AAV‐Ccl7^‐KD^ septic mice (n = 6). J) Proportion of CCR1^+^ IMs in the lung tissue of sham and CLP mice (n = 6). K, L) Proportions of the CD86^+^ M1 macrophages (K) or CD206^+^ M2 macrophages (L) among the CCR1^+^ IMs in the lung tissue of sham and CLP mice (n = 6). M) Representative immunofluorescence co‐localization of CCR1 (green) and CD86 (red) in lung sections from sham and CLP mice. The numbers of double‐positive (CCR1^+^CD86^+^) cells per field were quantified (arrows indicate double‐positive cells; scale bars: 50 µm, n = 6). N, O) Proportions of the CD86^+^ M1 macrophages (N) or CD206^+^ M2 macrophages (O) among the CCR1^+^ IMs in the lung tissue of AAV‐vector and AAV‐Ccl7^KD^ mice with or without sepsis (n = 6). P) Concentrations of IL‐1β, IL‐6, and TNF‐α in the BALF of AAV‐vector and AAV‐Ccl7^‐KD^ mice with or without sepsis (n = 6). Data are presented as mean ± SD, ns, nonsignificant. ^*^ *p* < 0.05, ^**^ *p* < 0.01, ^***^ *p* < 0.001. Data in A, E, and G‐M were analyzed by two‐tailed Student's *t*‐test. Data in B were analyzed by the Wilcoxon rank sum test. Data in N‐P were analyzed by two‐way ANOVA with Tukey's post hoc test.

Numerous studies have demonstrated that neutrophils and macrophages play crucial roles in the early stages of septic ALI.^[^ ^26^ ^]^ Clinical data from septic patients suggested that high CCL7 expression did not have a significant effect on neutrophil infiltration (**Figure** **2**A). Consequently, we utilized previously obtained RNA sequencing data to predict immune cell infiltration and observed a positive correlation between CCL7 expression and M1 macrophage infiltration (Figure 2B). Single‐cell sequencing data further confirmed the presence of numerous monocyte‐derived macrophages predominantly exhibiting the M1 phenotype in the lung tissue of CLP mice (Figure 2C).

Flow cytometry analysis of mouse lung tissue was conducted, and infiltrating macrophages (IMs) and alveolar macrophages (AMs) were sorted (Figure S3A, Supporting Information). The results confirmed that the proportions of total macrophages (TMs) and IMs increased under septic conditions (Figure S3B, Supporting Information). Immunohistochemical staining for CD31, CD86, and CCL7 was performed to verify the relationship between local endothelial CCL7 expression and M1 macrophage infiltration (Figure 2D). Areas with high endothelial CCL7 expression exhibited significant recruitment of M1 macrophages (Figure 2E). The intensity of CCL7 staining was positively correlated with the intensity of CD86 staining (Figure 2F). We then depleted and reinfused macrophages and further validated the role of CCL7 in mediating IMs. A macrophage scavenging agent (clodronate liposomes, CLs) was injected into the tail vein of mice for two days. Next, exogenous bone marrow‐derived macrophages (BMDMs) labeled with PKH26 were infused into the CLP mice (Figure S3C, D, Supporting Information). The infiltration of **PKH26‐labeled BMDMs** was reduced in the AAV‐**Ccl7^‐KD^** septic mice (Figure 2G). Additionally, the results revealed alleviated lung inflammation and partly increased lung volume in the AAV‐**Ccl7^‐KD^** septic mice (Figure S3E, F, Supporting Information). Pulmonary vascular permeability was also decreased in AAV‐**Ccl7^‐KD^** septic mice (Figure S3G, Supporting Information), and the levels of inflammatory cytokines in the BALF, including IL‐1β, IL‐6, and TNF‐α, were reduced (Figure S3H, Supporting Information), which was correlated with the alleviation of pathological lung tissue damage (Figure S3I, J, Supporting Information). Flow cytometry analysis demonstrated a significant reduction in the infiltration ratio of **PKH26‐labeled M1 macrophages** in lung tissue of the AAV‐**Ccl7^‐KD^** septic mice (Figure 2H), whereas the infiltration ratio of **PKH26‐labeled M2 macrophages** remained unaffected (Figure 2I). Furthermore, RNA sequencing analysis was conducted on selected **PKH26‐labeled** BMDMs. Kyoto Encyclopedia of Genes and Genomes (KEGG) enrichment analysis revealed that focal adhesion, leukocyte transendothelial migration, and cell adhesion molecule functions of **PKH26‐labeled** BMDMs were significantly downregulated in AAV‐Ccl7**^‐^** ^KD^ septic mice (Figure S3K, Supporting Information).

Previous studies have shown that CCL7 can bind to specific ligands, including CCR1, CCR2, and CCR3, which are involved in the recruitment of macrophages.^[^ ^27^, ^28^, ^29^ ^]^ Flow cytometry confirmed the recruitment of CCR1⁺ and CCR2⁺ IMs in the lungs of septic mice (Figure 2J, Figure S4A, Supporting Information). Single‐cell sequencing data revealed high expression of CCR1 on the surface of infiltrating M1 macrophages (Figure S4B, Supporting Information). Flow cytometry analysis confirmed that CCR2⁺ macrophages did not significantly polarize to the M1 phenotype (Figure S4C, Supporting Information), nor did CCR3⁺ macrophages (Figure S4D, Supporting Information). Instead, septic mice exhibited a significant increase in the number of CCR1⁺ M1 macrophages (Figure 2K), accompanied by a decreased proportion of CCR1⁺ M2 macrophages in the lung tissue (Figure 2L). Immunofluorescence experiments further confirmed the increased infiltration of CCR1⁺ M1 macrophages in septic mice (Figure 2M). Next, we investigated the effect of endothelial CCL7 expression on the function of IMs. Flow cytometry results demonstrated that specific inhibition of endothelial‐derived CCL7 effectively reduced the number of CCR1⁺ IMs (Figure S4E, Supporting Information), particularly CCR1⁺ M1 macrophages (Figure 2N), and increased the percentage of CCR1⁺ M2 macrophages instead (Figure 2O). However, there was no significant effect on the number of CCR2^+^ or CCR3^+^ IMs (Figure S4F, G, Supporting Information). Similarly, expression levels of IL‐1β, IL‐6, and TNF‐α in the BALF of AAV‐Ccl7^‐KD^ septic mice were lower than those in the BALF of AAV‐vector septic mice (Figure 2P). These findings suggest that specific inhibition of endothelial‐derived CCL7 delays the infiltration of CCR1^+^ macrophages into lung tissue and prevents their transformation to the pro‐inflammatory phenotype.

Subsequently, we explored the effect of CCR1⁺ macrophages on ECs by sorting CCR1^‐^ and CCR1⁺ macrophages and co‐culturing them directly with ECs (Figure S5A, Supporting Information). Principal component analysis (PCA) and GO enrichment analysis revealed that ECs co‐cultured with CCR1⁺ macrophages exhibited greater heterogeneity (Figure S5B, C, Supporting Information) and increased cell adhesion and damage (Figure S5D, Supporting Information). Furthermore, the expression of inflammatory cytokines (IL‐1α, IL‐1β, IL‐6, and CXCL16) and cell adhesion molecules (CD44, CDH11, ADAMTS4, and NECTIN1) in ECs were significantly upregulated. Conversely, the expression of the EC function marker CD34 was markedly downregulated. Additionally, markers indicative of endothelial damage, such as SNAI1, DAPK2, and COL1A1, were notably elevated (Figure S5E, Supporting Information). These findings collectively suggest that CCL7‐recruited CCR1⁺ macrophages exacerbate endothelial dysfunction during sepsis by promoting the production of inflammatory cytokines, enhancing cell adhesion, and inducing endothelial injury.

### 2.3 CCR1^+^ Macrophages Play a Crucial Role in Septic ALI

**Figure 3:**
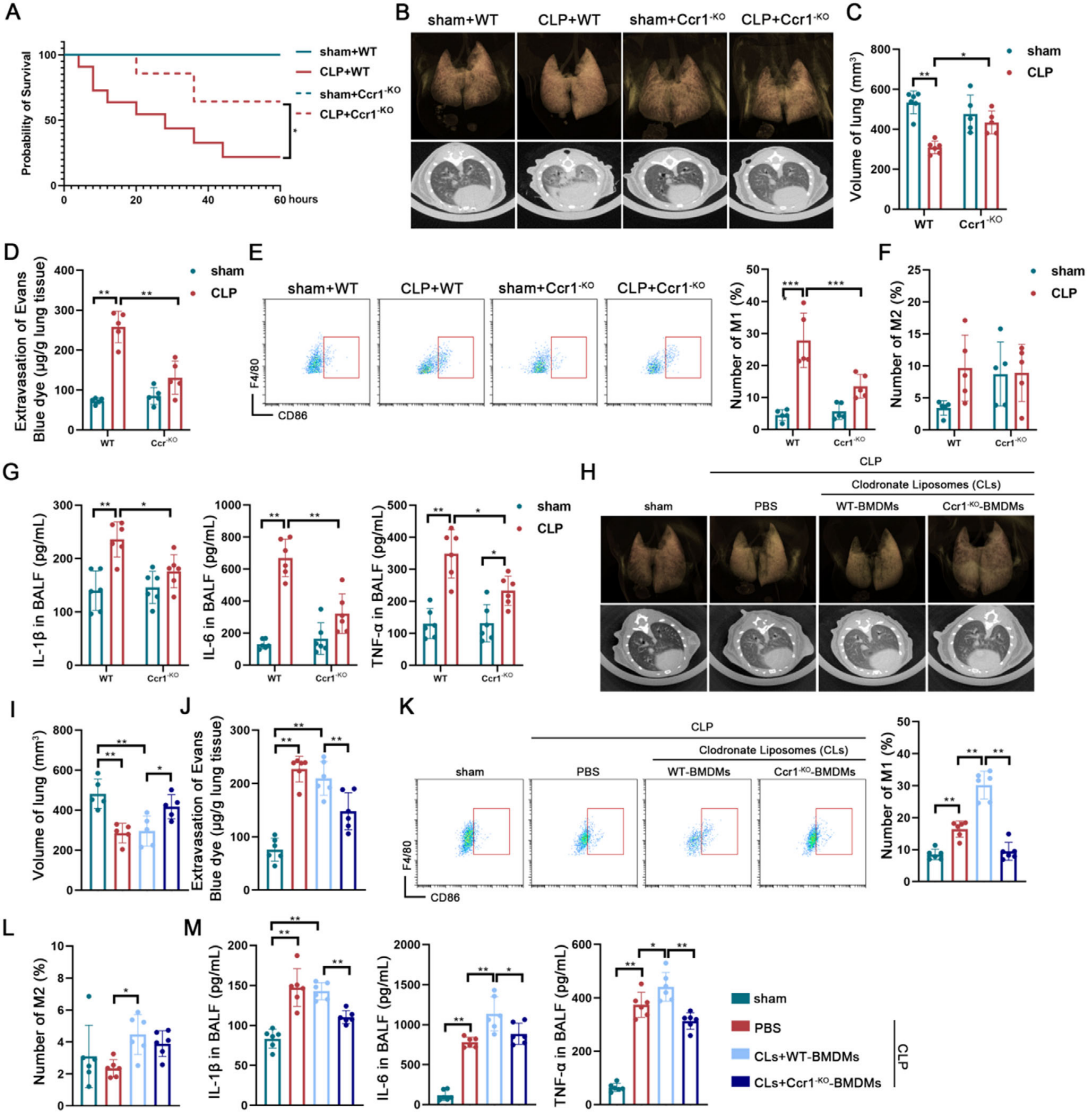
CCR1^+^ macrophages play a crucial role in septic ALI. A) Kaplan‐Meier survival curve comparing WT and Ccr1^‐KO^ mice with or without sepsis (n = 10). B) 3D imaging and CT scans of lungs from WT and Ccr1^‐KO^ mice with or without sepsis. C) Quantification of micro‐CT‐derived non‐aerated lung volume as an indicator of lung consolidation (n = 5/6). D) Assessment of lung transvascular permeability by measuring Evans blue dye leakage in micrograms per gram of lung tissue in WT and Ccr1^‐KO^ mice with or without sepsis (n = 5). E, F) Proportions of the CD86^+^ M1 macrophages (E) or CD206^+^ M2 macrophages (F) among the IMs in lung tissue of WT and Ccr1^‐KO^ mice with or without sepsis (n = 5). G) Concentrations of IL‐1β, IL‐6, and TNF‐α in the BALF of WT and Ccr1^‐KO^ mice with or without sepsis (n = 6). H) 3D imaging and CT scans of lungs in the indicated groups. I) Quantification of micro‐CT‐derived non‐aerated lung volume as an indicator of lung consolidation (n = 5). J) Assessment of lung transvascular permeability by measuring Evans blue dye leakage in micrograms per gram of lung tissue in the indicated groups (n = 6). K, L) Proportions of the CD86^+^ M1 macrophages (K) or CD206^+^ M2 macrophages (L) among the IMs in the lung tissue of the indicated groups (n = 6). M) Concentrations of IL‐1β, IL‐6, and TNF‐α in the BALF of the indicated groups (n = 6). Data are presented as mean ± SD, ^*^ *p* < 0.05, ^**^ *p* < 0.01. Data in A were analyzed by the Log‐rank test. Data in C were analyzed by two‐way ANOVA with Scheffe's post hoc test. Data in D‐G were analyzed by two‐way ANOVA with Tukey's post hoc test. Data in I‐M were analyzed by one‐way ANOVA with Tukey's post hoc test.

We generated Ccr1‐knockout (**Ccr1^‐KO^**) mice to examine the impact of **CCR1** deficiency on macrophage function (Figure S6A–C, Supporting Information). Survival analysis revealed that compared with wild‐type (WT) septic mice, Ccr1^‐KO^ septic mice presented a significantly lower mortality rate (**Figure** **3**A). Chest CT imaging revealed that **Ccr1^‐KO^** septic mice had milder pulmonary inflammation (Figure 3B) and partial restoration of sepsis‐induced lung volume reduction (Figure 3C). Pulmonary vascular permeability was also markedly lower in **Ccr1^‐KO^** septic mice than in WT septic mice (Figure 3D). Pathological analysis confirmed that the severity of lung injury was alleviated in **Ccr1^‐KO^** septic mice (Figure S6D, E, Supporting Information), accompanied by a significant reduction in endothelial cell apoptosis (Figure S6F, Supporting Information). Furthermore, lung tissue analysis revealed decreased infiltration of **M1 macrophages** in **Ccr1^‐KO^** septic mice (Figure 3E), whereas **M2 macrophages** remained unchanged (Figure 3F). The levels of inflammatory cytokines in the BALF were also significantly reduced **in Ccr1^‐KO^** septic mice (Figure 3G).

In addition, we conducted macrophage depletion and reinfusion experiments using BMDMs derived from WT and Ccr1^‐KO^ mice. Chest CT imaging revealed that CLP mice infused with WT BMDMs exhibited pulmonary inflammation and vascular leakage comparable to those in the CLP+PBS group (Figure 3H–J). Notably, M1 macrophage infiltration was significantly increased in the CLP+CLs+WT group (Figure 3K). In contrast, reinfusion of Ccr1^‐KO^ BMDMs resulted in increased lung volume and reduced vascular leakage compared with WT BMDMs (Figure 3H–J). M1 macrophage infiltration was significantly reduced in the CLP+CLs+ Ccr1^‐KO^ group (Figure 3K), and M2 macrophage infiltration was partially decreased (Figure 3L). Moreover, the CLP+CLs+Ccr1^‐KO^ group also exhibited significantly attenuated lung injury and reduced inflammatory cytokine secretion compared with those of the CLP+CLs+WT group (Figure 3M, Figure S6G–I, Supporting Information). In conclusion, these findings confirm that the increased infiltration of CCR1^+^ macrophages into lung tissue plays a pivotal role in sepsis‐induced lung injury. The CCL7–CCR1 axis represents a key mechanism mediating macrophages in the progression of septic ALI.

### 2.4 The CCL7–CCR1 Axis Regulates Macrophage Polarization via STAT1 Activation

**Figure 4:**
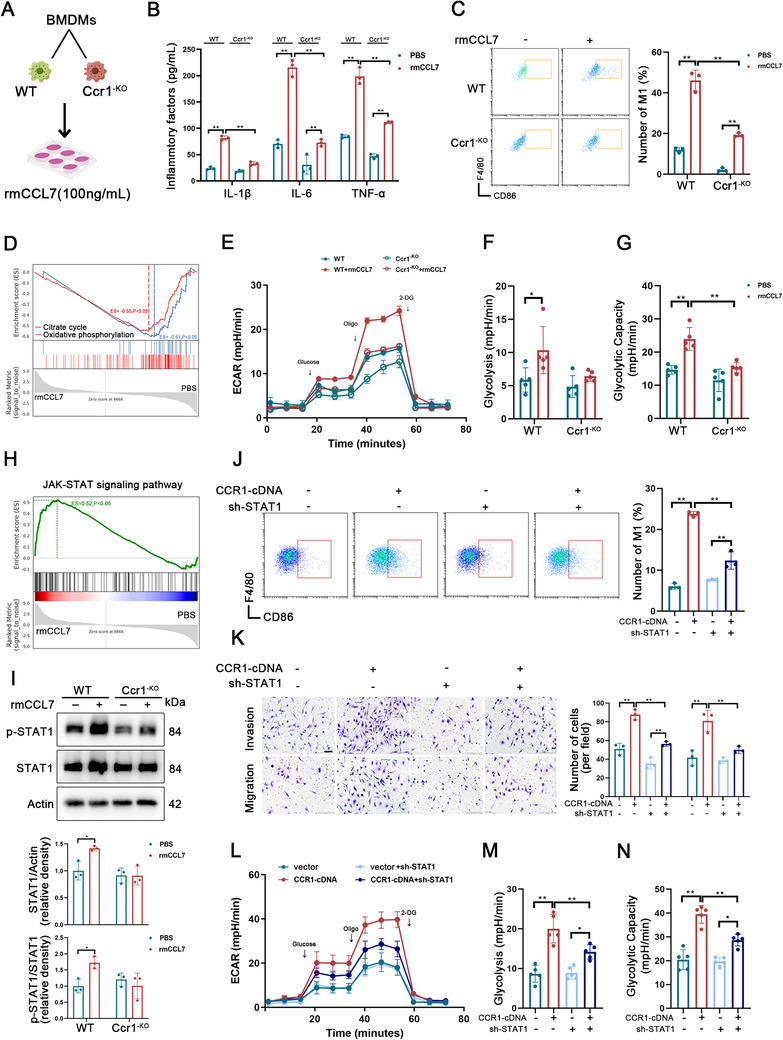
The CCL7–CCR1 axis regulates macrophage polarization via STAT1 activation. A) WT and Ccr1^‐KO^ BMDMs were treated with or without rmCCL7 (100 ng mL^−1^) for 24 h. B) Concentrations of IL‐1β, IL‐6, and TNF‐α in the supernatants of WT and Ccr1^KO^ BMDMs treated with or without rmCCL7 (n = 3). C) Proportions of the M1 macrophages among the WT and Ccr1^‐KO^ BMDMs treated with or without rmCCL7 (n = 3). D) GSEA was performed on rmCCL7‐treated BMDMs, revealing enrichment scores (ESs) for the citrate cycle and oxidative phosphorylation pathways. E‐G) ECAR in WT and Ccr1^KO^ BMDMs with or without rmCCL7 (n = 5). H) GSEA was conducted on rmCCL7‐treated BMDMs, revealing ES for the JAK‐STAT signaling pathway. I) Western blot analysis of STAT1 and p‐STAT1 expression in WT and Ccr1^KO^ BMDMs treated with or without rmCCL7. The values were normalized to those of the control group (n = 3). J) Proportion of the M1 macrophages in the indicated groups (n = 3). K) Invasion and migration abilities of BMDMs in the indicated groups (scale bar: 100µm, n = 3). L‐N) ECAR in the indicated groups (n=5). Data are presented as mean ± SD, ^*^ *p* < 0.05, ^**^ *p* < 0.01. Data in B, C, F, G, and I were analyzed by two‐way ANOVA with Tukey's post hoc test. Data in J, K, M, and N were analyzed by one‐way ANOVA with Tukey's post hoc test.

To investigate the functional role of the CCL7–CCR1 axis, we extracted conditioned medium (CM) from ECs and co‐cultured them with BMDMs (Figure S7A, Supporting Information). Flow cytometry analysis revealed that siRNA‐mediated inhibition of CCL7 secretion reversed the CM‐induced M1 polarization of BMDMs (Figure S7B, Supporting Information), whereas M2 polarization remained unaffected (Figure S7C, Supporting Information). Next, we cultured BMDMs with the exogenous recombinant mouse CCL7 protein (rmCCL7) (**Figure** **4**A). The rmCCL7‐treated BMDMs promoted the secretion of proinflammatory cytokines (Figure 4B), enhanced M1 polarization (Figure 4C), and increased migration and invasion of BMDMs (Figure S7D, Supporting Information). However, in Ccr1^‐KO^ BMDMs, rmCCL7 partially lost its effectiveness (Figure 4B, C; Figure S7D, Supporting Information). RNA sequencing analysis was performed to elucidate the molecular mechanism underlying the CCL7‒CCR1 functional axis. The results revealed that rmCCL7 upregulated M1‐associated genes and downregulated M2‐associated genes in BMDMs (Figure S7E, F, Supporting Information). Given that pro‐inflammatory macrophages primarily rely on glycolysis for energy metabolism^[^ ^30^ ^]^, GESA further revealed that rmCCL7 inhibited the citrate cycle and oxidative phosphorylation pathways (Figure 4D). In the aforementioned experiments, we confirmed that glycolysis/gluconeogenesis in PKH26**‐labeled** macrophages from AAV‐Ccl7^‐KD^ septic mice was decreased (Figure S5C, Supporting Information). These findings suggest that the CCL7‒CCR1 functional axis may be involved in regulating the metabolic reprogramming of macrophages. The seahorse assay confirmed that activation of the CCL7‒CCR1 axis increased glycolysis while suppressing oxidative phosphorylation in BMDMs (Figure 4E–G, Figure S7G–I, Supporting Information).

Further investigation into the mechanism revealed that CCR1 receptor activation upregulated JAK‐STAT pathway activity (Figure 4H). Western blot analysis confirmed that CCR1 receptor activation influenced the expression and phosphorylation levels of STAT1 (p‐STAT1) (Figure 4I). We then conducted inhibition and rescue assays. CCR1 cDNA was reintroduced into Ccr1^‐KO^ BMDMs, which partially restored pro‐inflammatory activation, migration, and invasion of the BMDMs. However, these effects were abolished upon STAT1 depletion (Figure 4J, K). Additionally, in CCR1‐overexpressing BMDMs, the trend of glycolysis was completely reversed when STAT1 was inhibited (Figure 4L,M). This phenomenon was accompanied by corresponding changes in oxidative phosphorylation (Figure S7J–L, Supporting Information). Therefore, we attempted to further verify the potential mechanism by which the CCL7‒CCR1 axis activated the JAK‐STAT pathway. However, as a regulator of STAT1 phosphorylation, activation of the CCL7‒CCR1 axis had no significant effect either on the expression or phosphorylation of JAK1/JAK2 or on the mRNA level of STAT1 (Figure S7M, N, Supporting Information). Thus, other posttranscriptional modifications may be involved in influencing the function of STAT1.

### 2.5 Metabolic Reprogramming in Macrophages is Dependent on STAT1^‐K665^ Succinylation

**Figure 5:**
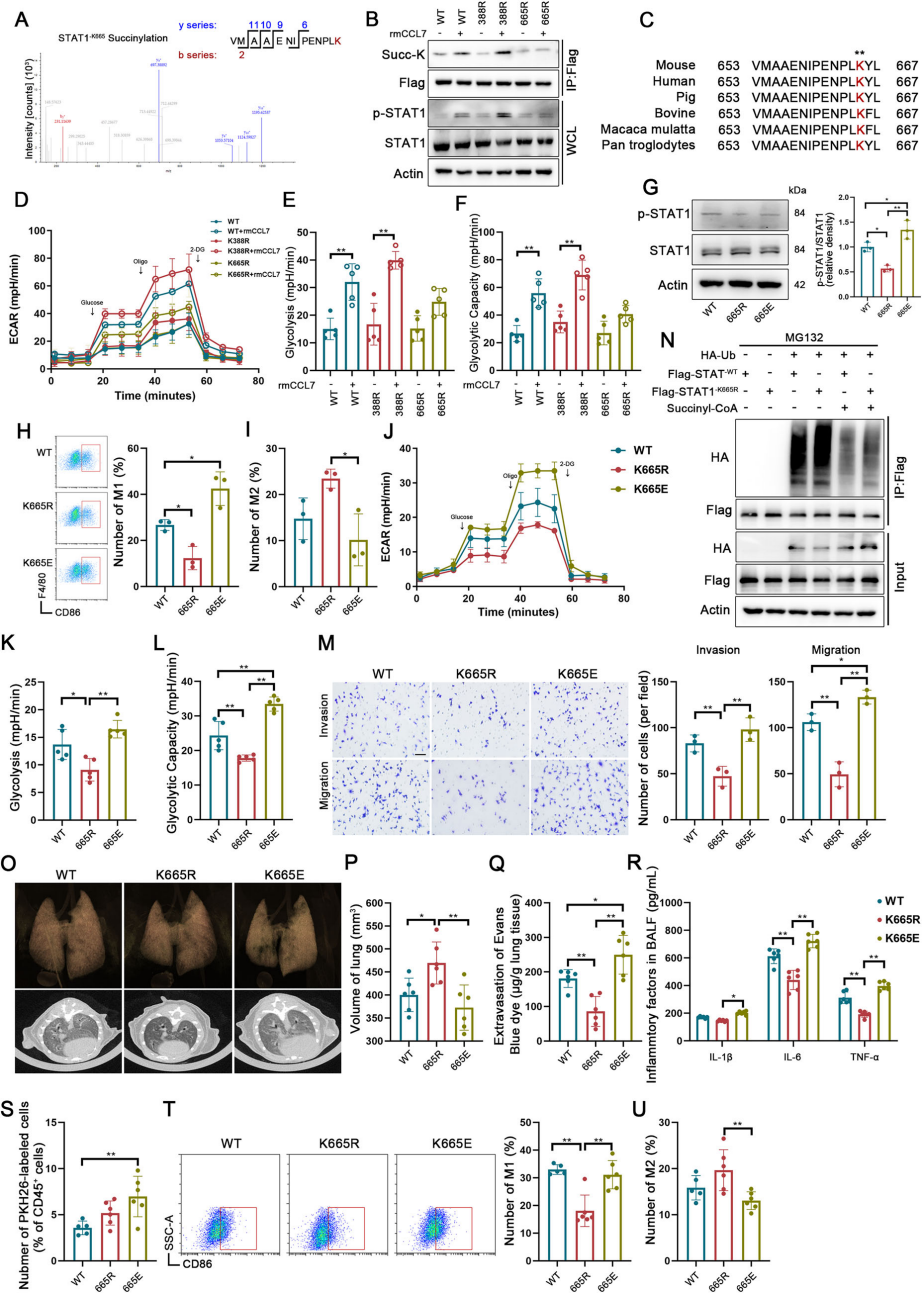
Metabolic reprogramming in macrophages is dependent on STAT1^‐K665^ succinylation. A) Lysine succinylation proteomics identifying the STAT1^‐K665^ modification site via LC‐MS/MS. B) Identification of STAT1 succinylation sites in BMDMs transfected with the STAT1^‐WT^, STAT1^‐K388R^, and STAT1^‐K665R^ plasmids. Succinylation was immunoprecipitated and detected by immunoblotting for Flag. C) Schematic representation of the STAT1 domain structure and alignment of STAT1 orthologs, with the succinylated residue (K665) highlighted in red. D‐F) ECAR in the STAT1^‐WT^, STAT1^‐K388R^, and STAT1^‐K665R^ BMDMs treated with or without rmCCL7 (n = 5). G) Western blot analysis of STAT1 and p‐STAT1 expression in STAT1^‐WT^, STAT1^‐K665R^, and STAT1^‐K665E^ BMDMs. The values were normalized to those of the STAT1^‐WT^ group (n = 3). H, I) Proportions of the CD86^+^ M1 macrophages (H) or CD206^+^ M2 macrophages (I) among the BMDMs in the STAT1^‐WT^, STAT1^‐K665R^, and STAT1^‐K665E^ groups (n = 3). J‐L) ECAR in the STAT1^‐WT^, STAT1^‐K665R^, and STAT1^‐K665E^ groups (n = 5). M) Invasion and migration abilities of BMDMs in the STAT1^‐WT^, STAT1^‐K665R^, and STAT1^‐K665E^ groups (scale bar: 100µm, n = 3). N) Immunoprecipitation analysis of STAT1 ubiquitination in HEK293T cells transiently transfected with HA‐ubiquitin (Ub), FLAG‐STAT1^‐WT^, and Flag‐STAT1^‐K665R^, with or without succinyl‐CoA treatment. O) 3D imaging and CT scans of lungs from septic mice transfused with the STAT1^‐WT^, STAT1^‐K665R^, and STAT1^‐K665E^ groups. P) Quantification of micro‐CT‐derived non‐aerated lung volume as an indicator of lung consolidation volume (n = 6). Q) Assessment of lung transvascular permeability by measuring Evans blue dye leakage in micrograms per gram of lung tissue in the STAT1^‐WT^, STAT1^‐K665R^, and STAT1^‐K665E^ groups (n = 6). R) Concentrations of IL‐1β, IL‐6, and TNF‐α in the BALF of septic mice transfused with the STAT1^‐WT^, STAT1^‐K665R^, and STAT1^‐K665E^ BMDMs (n = 6). S. Proportions of the PKH26‐labeled BMDMs in the lung tissue of septic mice transfused with the STAT1^‐WT^, STAT1^‐K665R^, and STAT1^‐K665E^ BMDMs (n = 5/6). T, U) Proportions of the CD86^+^ M1 macrophages (T) or CD206^+^ M2 macrophages (U) among the PKH26‐labeled BMDMs in the lung tissue of septic mice transfused with the STAT1^‐WT^, STAT1^‐K665R^, and STAT1^‐K665E^ BMDMs (n = 5/6). Data are presented as mean ± SD, ^*^ *p* < 0.05, ^**^ *p* < 0.01. Data in E‐I, K, M, and P‐R were analyzed by one‐way ANOVA with Tukey's post hoc test. Data in S‐U were analyzed by one‐way ANOVA with Scheffe's post hoc test.

Previous studies have established that posttranscriptional modification plays a crucial role in macrophage function. Western blot analysis confirmed that the CCL7–CCR1 axis modulated intracellular succinylation, but not methylation or acetylation in BMDMs (Figure S8A, Supporting Information). We investigated the effects of exogenous succinyl‐CoA, which induced an increase in STAT1 succinylation in a concentration‐dependent manner, thereby altering the corresponding expression of p‐STAT1 (Figure S8B, Supporting Information). To further elucidate the specific role of STAT1 succinylation, we performed liquid chromatography tandem‐mass spectrometry (LC‐MS/MS), which identified lysine residues K388 and K665 of STAT1 as potential succinylation sites (**Figures** **5**A, S8C, Supporting Information). Mutant plasmids (K‐to‐R, mimicking hypo‐succinylation) were generated to validate the functional significance of these sites. Western blot analysis revealed that STAT1^‐K388R^ had no effect on rmCCL7‐induced succinylation, whereas STAT1^‐K665R^ significantly reduced STAT1 succinylation, leading to a decrease in p‐STAT1 expression (Figure 5B). Notably, the K665 site is highly conserved across other species (Figure 5C). Functionally, even upon activation of the CCL7–CCR1 axis, STAT1^‐K665R^ markedly suppressed the expression of inflammatory cytokines (Figure S8D–F, Supporting Information), inhibited glycolysis in BMDMs (Figure 5D–F), and promoted oxidative phosphorylation (Figure S8G–I, Supporting Information).

We subsequently generated a hyper‐succinylation mutant (K665E) to further validate the specificity of K665 succinylation in modulating STAT1 function. Compared with STAT1^‐WT^ BMDMs, STAT1^‐K665E^ presented significantly elevated p‐STAT1 levels (Figure 5G), promoted macrophage polarization toward the M1 phenotype (Figure 5H), and suppressed M2 polarization (Figure 5I). Moreover, glycolysis was markedly increased in STAT1^‐K665E^ BMDMs (Figure 5J–L), while oxidative phosphorylation was attenuated (Figure S8J–L, Supporting Information). Functionally, the STAT1^‐K665R^ mutation impaired macrophage migration and invasion, whereas STAT1^‐K665E^ reversed these effects (Figure 5M). The recent studies have reported that succinylation modification inhibits ubiquitination and degradation of proteins.^[^ ^31^, ^32^ ^]^ Thus, we investigated the ubiquitination status of STAT1. STAT1^‐K665R^ mutation resulted in increased STAT1 ubiquitination, counteracting succinyl‐CoA‐induced suppression of ubiquitination partly (Figure 5N). This might explain how succinylation leads to the functional change of STAT1.

In vivo, we overexpressed STAT1^‐WT^, STAT1^‐K665R^, and STAT1^‐K665E^ in BMDMs and reinfused them into septic mice as previously described. Chest CT analysis revealed that, compared with that in the STAT1^‐WT^ group, the lung inflammatory response was attenuated in the STAT1^‐K665R^ group but exacerbated in the STAT1^‐K665E^ group (Figure 5O). Similarly, the lung volume was partially restored in the STAT1^‐K665R^ group but significantly worsened in the STAT1^‐K665E^ group (Figure 5P), with pulmonary vascular permeability exhibiting comparable trends (Figure 5Q). Analysis of the BALF further confirmed that inflammatory cytokine levels were markedly reduced in the STAT1^‐K665R^ group, whereas they were elevated in the STAT1^‐K665E^ group (Figure 5R). Compared with the STAT1^‐WT^ group, the STAT1^‐K665E^ group exhibited increased infiltration of PKH26‐labeled macrophages (Figure 5S), accompanied by enhanced M1 polarization and reduced M2 polarization (Figure 5T, U), the STAT1^‐K665R^ group presented the opposite trends.

### 2.6 The CCL7–CCR1 Axis Upregulates KAT2A to Drive STAT1 Succinylation

**Figure 6:**
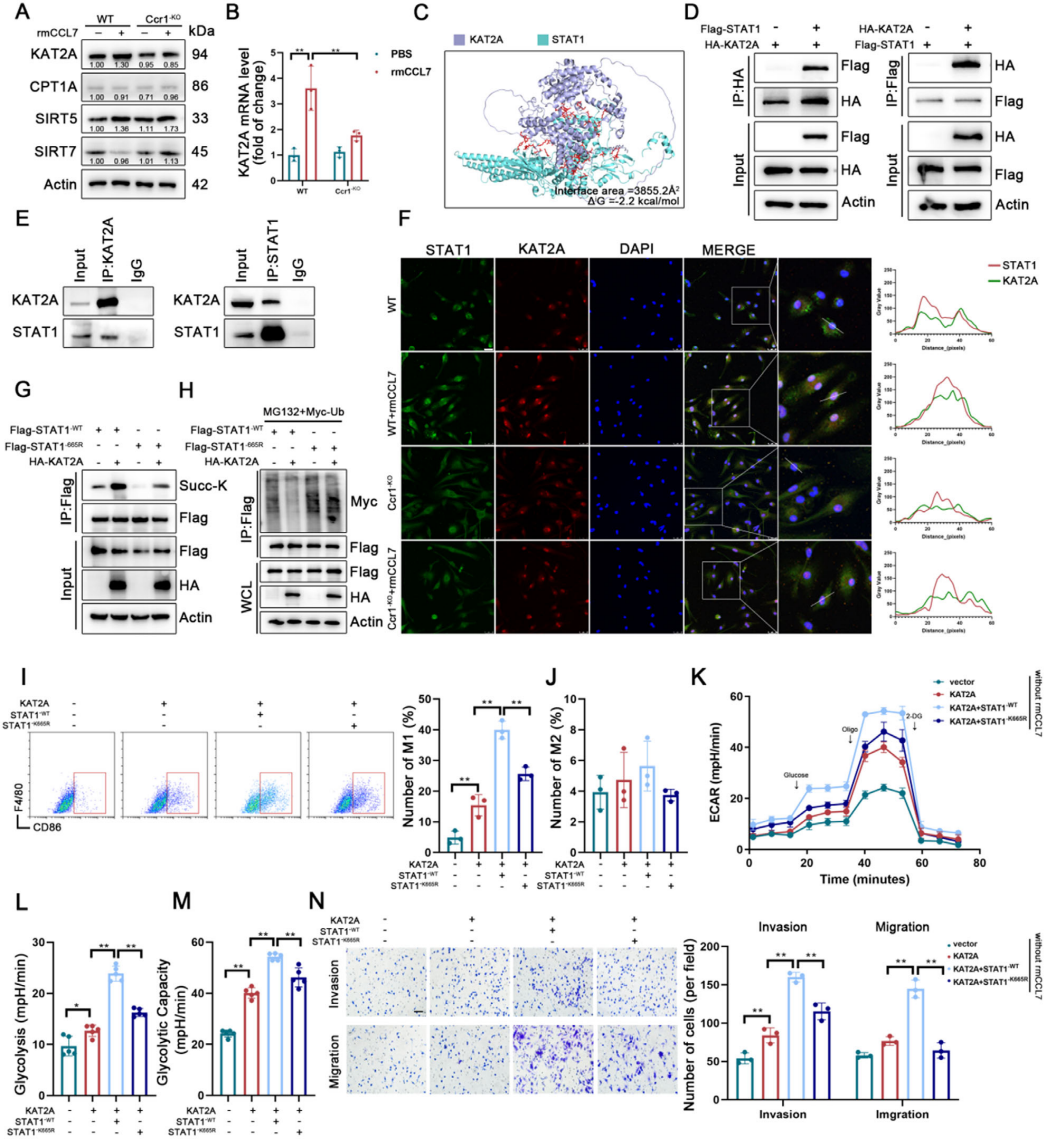
The CCL7–CCR1 axis upregulates KAT2A to drive STAT1 succinylation. A) Western blot analysis of KAT2A, CPT1A, SIRT5, and SIRT7 protein levels in WT and Ccr1^‐KO^ BMDMs treated with or without rmCCL7 for 24 h. B) Relative mRNA expression of KAT2A in WT and Ccr1^‐KO^ BMDMs treated with or without rmCCL7 for 24 h (n = 3). C) Structural model of KAT2A (purple) binding to STAT1 (blue), highlighting key interacting residues (red sticks) and the interface area (3855.2 Å^2^). The predicted binding energy (Δ_i_G = −2.2 kcal mol^−1^) indicated a stable interaction. D) Interaction between HA‐tagged KAT2A and Flag‐tagged STAT1 was assessed in HEK293T cells co‐transfected to overexpress these proteins. The interaction was analyzed in whole cell lysates (WCLs) and after immunoprecipitation with anti‐Flag or anti‐HA beads. E) Endogenous interaction between KAT2A and STAT1 was determined by co‐immunoprecipitation in BMDM lysates. F) Representative immunofluorescence images of STAT1 (red) and KAT2A (green) localization in the WT and Ccr1^‐KO^ BMDMs treated with or without rmCCL7 for 24 h. The right panel showed the results of the co‐localization analysis (scale bars: 50 µm). G) Immunoblot analysis of succinylated STAT1 in HEK293T cells transfected with Flag‐STAT1^‐WT^ or Flag‐STAT1^‐K665R^, with or without HA‐KAT2A. H) Immunoblot analysis of ubiquitinated STAT1 in HEK293T cells transfected with Flag‐STAT1^‐WT^ or Flag‐STAT1^‐K665R^, with or without HA‐KAT2A. I, J) Proportion of the CD86^+^ M1 macrophages (I) or CD206^+^ M2 macrophages (J) among the BMDMs in the indicated groups (n = 3). K‐M) ECAR in the indicated groups (n = 5). N) Invasion and migration abilities of BMDMs in the indicated groups (scale bar: 100µm, n = 3). Data are presented as mean ± SD, ^*^ *p* < 0.05, ^**^ *p* < 0.01. Data in I, J, and L‐N were analyzed by one‐way ANOVA with Tukey's post hoc test.

The primary enzymes known to regulate protein succinylation include KAT2A, CPT1A, SIRT5, and SIRT7.^[^ ^33^ ^]^ Western blot and qPCR analyses revealed that KAT2A expression was regulated by the CCL7–CCR1 axis (**Figure** **6**A, B). Previous studies have reported that KAT2A plays a critical role in macrophage proinflammatory responses.^[^ ^23^ ^]^ Consistent with these findings, we observed that silencing KAT2A reversed rmCCL7‐induced M1 polarization (Figure S9A–D, Supporting Information), facilitated metabolic shift from glycolysis to oxidative phosphorylation (Figure S9E–J, Supporting Information), and inhibited cell migration and invasion (Figure S9K, Supporting Information).

We employed molecular docking models to determine whether KAT2A contributes to macrophage function through STAT1 succinylation. The results revealed a potential binding interaction between KAT2A and STAT1 (Figure 6C, Supplementary Table S1). This interaction was further validated via both exogenous and endogenous co‐immunoprecipitation assays (Figure 6D, E). Immunofluorescence staining also confirmed the co‐localization of STAT1 and KAT2A upon activation of the CCL7‐CCR1 axis (Figure 6F). Additionally, IP experiments confirmed the involvement of KAT2A in the succinylation of the STAT1^‐K665^ site (Figure 6G). The results of the ubiquitination assay also demonstrated that KAT2A reduced ubiquitination of STAT1 (Figure 6H).

Through inhibition and rescue experiments, we observed that overexpression of KAT2A in BMDMs significantly enhanced M1 polarization (Figure 6I, J) and promoted metabolic reprogramming, even in the absence of rmCCL7 stimulation (Figure 6K–M, Figure S9L–N, Supporting Information), which also affected migration and invasion of macrophages (Figure 6N). Moreover, in KAT2A‐overexpressing BMDMs, STAT1^‐WT^ further augmented pro‐inflammatory activation and metabolic reprogramming, whereas overexpression of STAT1^‐K665R^ inhibited these changes (Figure 6I‐N). These findings suggest that CCL7–CCR1 activation upregulates KAT2A, driving STAT1^‐K665^ succinylation and consequently contributing to macrophage polarization and metabolic reprogramming.

### 2.7 The Binding of STAT1 to the Promoter Region of Glycolysis‐Related Genes is Increased by Succinylation

**Figure 7:**
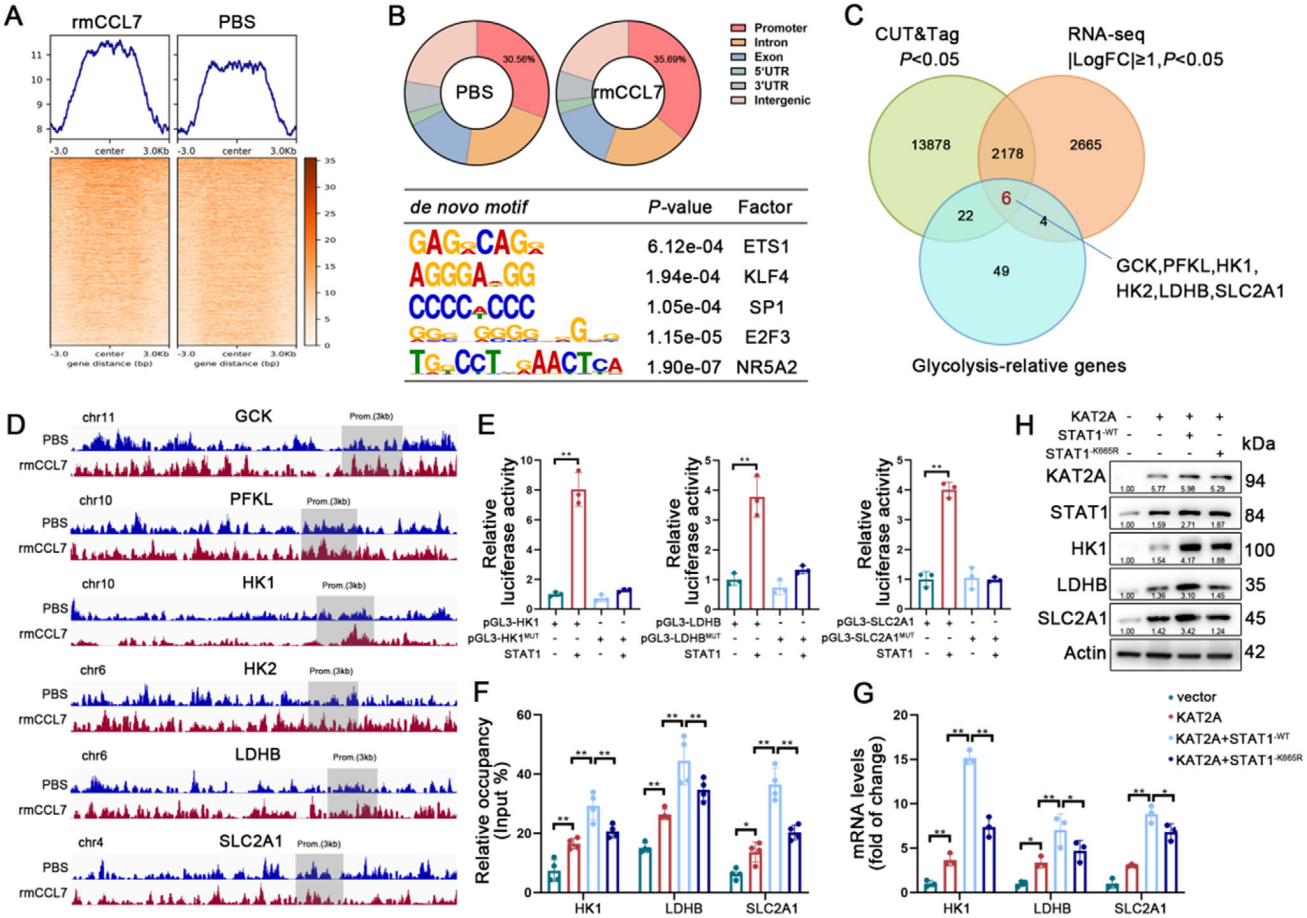
The binding of STAT1 to the promoter region of glycolytic genes is increased by succinylation. A) Heat map visualization of STAT1 CUT&Tag signal intensity profiles in between the BMDMs treated with or without rmCCL7, ranked by the magnitude of differential chromatin accessibility peaks. B) Genomic annotations of peaks by chromosome region (upper). The discovered motifs are enriched in the promoters of glycolysis‐related transcription factors (lower). C) Overlap analysis of differentially enriched STAT1‐binding peaks, differentially expressed genes in BMDMs treated with or without rmCCL7, and glycolysis‐associated gene clusters. D) Genome browser tracks illustrating STAT1 transcriptional binding patterns at loci of glycolytic enzymes (GCK, PFKL, HK1, HK2, LDHB, and SLC2A1) in BMDMs treated with or without rmCCL7. Promoter regions are demarcated by gray shading. E) Dual‐luciferase assay after the transfection with the indicated plasmids. F) Quantification of the abundance of STAT1 peaks at the promoters of glycolytic genes in the indicated groups (n = 3). G) Relative mRNA levels of HK1, LDHB, and SLC2A1 in the indicated groups (n = 3). H) Western blot analysis of HK1, LDHB, and SLC2A1 expression in the indicated groups. Data are presented as mean ± SD, ^*^ *p* < 0.05, ^**^ *p* < 0.01. Data in E‐G were analyzed by one‐way ANOVA with Tukey's post hoc test.

As a transcription factor, STAT1 plays a pivotal role in regulating the transcriptional efficiency of target genes. CUT&Tag analysis revealed that rmCCL7 enhanced STAT1 occupancy at promoter regions (**Figure** **7**A). Enrichment analysis revealed that STAT1 binding was predominantly localized to promoters, accounting for 35.69% of the total binding events (Figure 7B, upper panel). Furthermore, identified motifs were associated with several glycolysis‐related transcription factors, including ETS1, KLF4, SP1, E2F3, and NR5A2 (Figure 7B, lower panel). Integration of CUT&Tag and RNA sequencing data with glycolysis‐related genes indicated that STAT1 contributed to macrophage metabolic reprogramming by modulating key glycolysis‐related genes, including GCK, PFKL, HK1, HK2, LDHB, and SLC2A1 (Figure 7C). Further visualization and peak annotation analyses demonstrated that rmCCL7 stimulation increased STAT1 binding at the promoter and transcriptional regions of genes (Figure 7D). The specific STAT1 binding sites were mapped (Figure S10A, Supporting Information), and ChIP‐PCR further confirmed the presence of STAT1 in the promoter regions of HK1, LDHB, and SLC2A1 (Figure S10B, Supporting Information). The dual‐luciferase reporter assay validated the precise STAT1‐binding sites that regulated HK1, LDHB, and SLC2A1 transcription (Figure 7E).

Additionally, we confirmed that the CCL7–CCR1 axis influenced STAT1 binding efficiency at the promoter regions of HK1, LDHB, and SLC2A1 (Figure S10C, Supporting Information), thereby modulating subsequent gene transcription and protein translation (Figure S10D, E, Supporting Information). In KAT2A‐overexpressing BMDMs, STAT1 binding at the promoter regions of HK1, LDHB, and SLC2A1 was significantly increased (Figure 7F), accompanied by the upregulation of corresponding mRNA and protein expression (Figure 7G, H). Furthermore, overexpression of STAT1^‐WT^ further increased the expression of these glycolytic genes, whereas this effect was abolished upon overexpression of STAT1^‐K665R^, indicating that KAT2A‐mediated succinylation of STAT1 at lysine residue 665 plays a critical role in metabolic reprogramming.

## 3 Discussion

During the progression of sepsis, pulmonary vascular endothelial cells secrete a variety of inflammatory factors and chemokines. This process triggers inflammatory cascades and serves as a critical driving force in the pathogenesis of septic ALI.^[^ ^34^ ^]^ Chemokine CCL7, also known as monocyte chemoattractant protein‐3 (MCP‐3), is predominantly secreted by endothelial cells in situ.^[^ ^35^ ^]^ Previous studies have shown that chemokine CCL7 is elevated in the serum of septic patients.^[^ ^36^, ^37^ ^]^ In the present study, we also confirm a significant increase of CCL7 in non‐surviving patients with sepsis, which is positively correlated with the mortality of septic patients. In mouse models, specific inhibition of endothelial‐derived CCL7 effectively alleviates pulmonary inflammation and reduces the mortality of septic mice.

The interactions between pulmonary vascular endothelial cells and immune cells represent a novel research direction in the field of septic ALI.^[^ ^6^ ^]^ Macrophages are the principal inflammatory cells involved in the pathogenesis of septic ALI.^[^ ^38^ ^]^ Endothelial‐derived CCL7 further amplifies the inflammatory response by recruiting macrophages.^[^ ^39^, ^40^, ^41^ ^]^ Chemokine CCL7 induces infiltration of macrophages to organs by binding to specific receptors CCR1, CCR2, and CCR3.^[^ ^10^, ^39^ ^]^ Studies have shown that CCR2, a marker on the surface of classic monocytes, is mainly regulated by CCL2.^[^ ^42^ ^]^ Our research confirms that CCL7 has no effect on the recruitment of macrophages via the CCR2 receptor. CCR3 is mainly expressed on the surface of eosinophils.^[^ ^43^ ^]^ Instead, CCL7 plays a pivotal role in the recruitment of CCR1^+^ macrophages into lung tissue and specifically induces M1 polarization of CCR1^+^ macrophages during sepsis. The inhibition of endothelial‐derived CCL7 significantly reduces CCR1^+^ M1 macrophage accumulation in lung tissues, mitigates lung inflammation, and partially restores vascular endothelial barrier function in septic mice. Both in vitro and in vivo data reveal that CCR1 deficiency markedly inhibits CCL7‐induced macrophage mobilization and inflammatory responses. These results suggest that the CCL7‐CCR1 axis is a potential biomarker and therapeutic target for septic ALI.

The CCL7–CCR1 functional axis regulates proinflammatory differentiation of macrophages via activation of pathways such as the NF‐κB and JAK‐STAT pathways.^[^ ^10^, ^44^ ^]^ In this study, we demonstrated that the CCL7–CCR1 axis promotes STAT1 activation in macrophages. Succinylation plays a crucial role in modulating protein properties, including subcellular localization, function, stability, and affinity for other molecules, and is involved in regulating innate immune response.^[^ ^45^, ^46^, ^47^ ^]^ Inhibiting succinylation in macrophages has been shown to suppress M1 polarization and reduce inflammatory cytokine secretion.^[^ ^48^ ^]^ Proteomic and succinylomic analyses have identified STAT1 succinylation as a novel therapeutic target for silicosis.^[^ ^19^ ^]^ Our results reveal that targeted modulation of STAT1 succinylation in macrophages represents a novel therapeutic strategy for clinical intervention in septic ALI. The studies have reported that ubiquitination suppresses STAT1 activity.^[^ ^49^, ^50^ ^]^ Succinylation modification inhibits ubiquitination and degradation of proteins.^[^ ^31^, ^32^ ^]^ We elucidate the interplay between succinylation and ubiquitination of STAT1, demonstrating that succinylation of the K665 site of STAT1 inhibits its ubiquitination, thereby maintaining protein activity. Hence, succinylation at the K665 site of STAT1, which is regulated by the CCL7–CCR1 axis, is crucial for macrophage transformation to the proinflammatory phenotype.

Macrophage metabolic reprogramming governs activation, with enhanced aerobic glycolysis driving the proinflammatory phenotype.^[^ ^51^ ^]^ Our research demonstrates that STAT1 succinylation plays an essential role in regulating metabolic reprogramming in macrophages. We employed CUT&Tag and ChIP‐qPCR to verify the molecular mechanism underlying this phenomenon. These results confirm that the activation of the CCL7–CCR1 axis modulates STAT1 binding efficiency at the promoter regions of key glycolytic genes, including HK1, LDHB, and SLC2A1, thereby regulating the transcriptional and translational progression of these genes. Given that the metabolic shift in M1 macrophages bypasses the TCA cycle, elevated succinate levels provide abundant substrates for concurrent succinylation modifications.^[^ ^51^, ^52^ ^]^ Furthermore, in STAT1^‐K665R^ BMDMs, STAT1 binding to the promoter regions of HK1, LDHB, and SLC2A1 was significantly diminished. These results reinforce the notion that succinylation at the K665 site of STAT1 is pivotal in regulating enhanced glycolysis in macrophages.

To date, the primary enzymes identified as regulators of succinylation modifications include KAT2A, CPT1A, SIRT5, and SIRT7.^[^ ^18^ ^]^ Our findings indicate that CCL7, through CCR1 receptor activation, specifically alters KAT2A expression in macrophages, thus participating in the succinylation of STAT1^‐K665^. Previous research has implicated KAT2A in the metabolic reprogramming of macrophages.^[^ ^23^ ^]^ We observed that knockdown of KAT2A in the presence of rmCCL7 inhibited the proinflammatory response of macrophages and promoted the metabolic shift toward oxidative phosphorylation, whereas KAT2A overexpression had the opposite effect. These results suggest that KAT2A is involved in the CCL7–CCR1 axis‐mediated metabolic reprogramming and proinflammatory regulation. Our research also reveals that KAT2A interacts with STAT1, regulating the balance between succinylation and ubiquitination of STAT1, thereby promoting STAT1 binding at glycolysis‐related gene promoters. This effect is abrogated by STAT1^‐K665^ hyposuccinylation.

**Figure 8:**
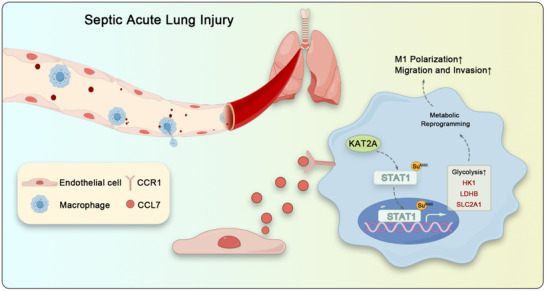
During sepsis, pulmonary vascular endothelial cells secrete chemokine CCL7, which plays a pivotal role in facilitating the infiltration of C‐C motif chemokine receptor 1‐positive (CCR1^+^) macrophages into lung tissue. Activation of the CCL7–CCR1 axis enhances the succinylation of STAT1 mediated by KAT2A, thereby driving metabolic reprogramming and promoting M1 polarization of macrophages. These findings elucidate the interaction between pulmonary vascular endothelial cells and macrophages in the pathogenesis of acute lung injury (ALI) during sepsis, offering novel insights and potential therapeutic targets for the treatment of septic ALI.

In conclusion, our study reveals that the CCL7–CCR1 axis increases KAT2A expression in macrophages, which mediates the succinylation of STAT1 at the K665 site to promote metabolic reprogramming. This mechanism enhances M1 polarization and infiltration of macrophages, contributing to the development of septic ALI (**Figure** **8**). Our study further elucidates the interaction between pulmonary vascular endothelial cells and macrophages, providing novel insights and potential therapeutic targets for the treatment of septic ALI.

### 3.1 Limitations of the Study

Several limitations of this study should be acknowledged. First, this study exclusively used male mice to control for hormonal variability, and we acknowledge gender‐based analysis as a current limitation. We need to plan follow‐up investigations in female mice to evaluate potential sex‐specific mechanisms. Second, although CCL7 is a key chemokine contributing to macrophage activation, the potential involvement of other immune cells and factors cannot be ruled out. In vivo experiments demonstrated that CCL7‐induced macrophage activation was abrogated in Ccr1^‐KO^ mice, highlighting the critical role of CCR1 receptor activation in sepsis‐related immune responses. Furthermore, given that immune cells such as eosinophils and dendritic cells are involved in the early immune response to sepsis and that their functions are also regulated by CCR1, the roles of these cell types in septic ALI warrant further investigation. Future studies should address these limitations by generating conditional knockout mice with CCR1‐specific depletion in macrophages, thereby enabling a more comprehensive understanding of the relationships among the CCL7–CCR1 axis, STAT1 succinylation, and septic ALI.

## 4 Experimental Section

The key resources used are listed in Table S2 (Supporting Information).

### Ethical Declaration

This study was approved by the Ethics Committee of Zhongshan Hospital, Fudan University (B2021‐182R). Written informed consent was obtained from all participants or their legal representatives before sample collection. All mouse experiments were approved by the Animal Review Committee of Zhongshan Hospital, Fudan University (2020‐119).

### Mouse Primary Endothelial Cells (ECs)

Mouse primary endothelial cells were isolated and cultured following the previously established protocol.^[^ ^53^ ^]^ Specifically, 6‐week‐old male C57BL/6 mice were deeply anesthetized with tribromoethanol (200 mg kg^−1^, intraperitoneal injection), after which their lungs were excised and rinsed with PBS supplemented with heparin. The lung tissues were then minced into 1 mm^3^ pieces and digested in the buffer containing collagenase IV (1 mg mL^−1^), dispase II (0.2 U mL^−1^), and DNase I (0.02 U mL^−1^) at 37 °C for 1 h. The resulting cell suspension was filtered and centrifuged at 1500 rpm for 5 min. The red blood cell lysis buffer was used to remove red blood cells for 5 min on ice. Subsequently, the isolated cells were incubated with 10 µg of CD45 or CD31 microbeads for 15 min at 4°C to facilitate magnetic‐activated cell sorting. Following sorting, the ECs were resuspended in the complete endothelial cell medium consisting of fetal bovine serum (FBS, 5%), endothelial cell growth supplement (ECGS, 1%), and penicillin‐streptomycin solution (P/S, 1%). Primary endothelial cells between passages 3 and 4 were utilized for subsequent experiments.

### Mouse Bone Marrow‐Derived Macrophages (BMDMs)

For the preparation of BMDMs, male 6‐week‐old C57BL/6 mice were deeply anesthetized with tribromoethanol (200 mg kg^−1^, intraperitoneal injection). Bone marrow cells were harvested from the femurs and tibias of the mice. Macrophage precursors were then cultured in Dulbecco's Modified Eagle's Medium (DMEM) supplemented with 10% FBS and 1% P/S, along with recombinant mouse M‐CSF (100 ng mL^−1^), to induce differentiation into mature macrophages for 6 days.

### Human Samples

Human blood samples were collected from healthy volunteers (n = 10) and patients with sepsis (n = 10) following the approved study protocol (B2021‐182R), which was authorized by the Ethics Committee of Zhongshan Hospital, Fudan University. Written informed consent was obtained from all participants or their legal representatives before sample collection.

### Mouse Cecal Ligation and Puncture (CLP) Model

Following randomization into experimental groups, mice were subjected to a 12‐h fasting period before surgery. Anesthesia was induced via the intraperitoneal injection of tribromoethanol (200 mg kg^−1^). Subsequently, the abdominal cavity was opened, and one‐half of the cecum was ligated via a 4‒0 suture and punctured twice with an 18‐gauge needle. A small amount of feces was gently extruded to ensure perforation, after which the peritoneum and skin were closed with interrupted sutures. Each animal received a subcutaneous injection of 0.5 mL of normal saline for rehydration. The mice in the sham group underwent identical surgical procedures without cecal ligation or puncture. After 24 h, the mice were euthanized, and BALF was collected via intratracheal injection of 1 mL of cold PBS, followed by three cycles of inflation and aspiration. Blood samples and lung tissues were harvested at the end of the modeling process for subsequent analyses. All mouse experiments were approved by the Animal Review Committee of Zhongshan Hospital, Fudan University (2020‐119).

### AAV6‐Mediated Silencing of Endothelial‐CCL7 in Mice

The AAV6 vector designed for silencing CCL7 and labeled with a green fluorescent protein (GFP) was constructed for this study. The vectors AAV6‐Tie2‐miR30‐shCcl7 and AAV6‐Tie2‐miR30‐shNC were administered via tracheal injection following protocols described in previous studies.^[^ ^54^, ^55^, ^56^ ^]^ Four weeks post‐injection, the CLP mouse model was established as outlined above.

### Flow Cytometry

Lung tissues were enzymatically digested and filtered through 70 µm cell strainers to generate single‐cell suspensions for analysis. The cells were incubated with an Fc blocker for 20 min to minimize non‐specific antibody binding. Subsequently, cells were stained with Zombie Aqua dye for viability assessment, followed by incubation with specific antibodies for 2 h at 4 °C in the dark. After staining, cells were washed twice with PBS. The following antibodies were utilized: anti‐CD45, anti‐CD11b, anti‐F4/80, anti‐SiglecF, anti‐CD86, anti‐CD206 and anti‐CCR1/CCR2/CCR3. Flow cytometry data were analyzed using CytExpert software.

### Micro‐CT and Imaging Analysis

The mice were anesthetized with isoflurane (2% oxygen) and positioned on a micro‐CT bed (Quantum GX2 MicroCT). A scout view was acquired with the following parameters: 90 kV, 88 µA, 115 mGy, and 72 µm voxel size. The mice were continuously monitored during scanning. Visualization and quantification of the images were performed using CT viewer software.

### CUT&Tag Analysis

The CUT&Tag analysis was performed using the HyperactiveTM In Situ ChIP Library Prep Kit for Illumina according to the manufacturer's instructions.^[^ ^57^ ^]^ Briefly, hyperactive pA‐Tn5 transposases were sequentially incubated with cells bound to the ConA beads. The hyperactive pA‐Tn5 transposase specifically cleaves DNA fragments associated with the target protein. The purified PCR products were evaluated using an Agilent 2100 Bioanalyzer. Libraries were sequenced on the Illumina NovaSeq 6000 platform, yielding 150 bp paired‐end reads for subsequent analysis. Different peaks between the rmCCL7 and PBS groups were identified using MAnorm software.^[^ ^58^ ^]^ First, a regression model was established for the M value and A value of the common peak between the two samples to account for sequencing bias. Subsequently, the Bayesian model was applied to calculate p‐values. Peaks with p < 0.05 and |M value| > 1 were considered significantly different.

### Single‐Cell Sequencing Analysis

Single‐cell sequencing data were obtained from the GEO database under accession number GSE207651. The count matrices and normalized expression matrices were processed using R (version 4.0.2). All datasets were merged, normalized, scaled, clustered, and visualized via t‐distributed stochastic neighbor embedding (t‐SNE). Marker genes for each cluster were identified using the “FindAllMarkers” function with default parameters. Functional enrichment analysis of GSEA pathways was performed using the “ClusterProfiler” package.

### RNA Sequencing and Analysis of Differentially Expressed Genes

Total RNA was extracted from cells using a TRIzol reagent. Transcriptome sequencing was conducted by the Illumina NovaSeq 6000 platform to generate 150 bp paired‐end reads. Raw reads in fastq format were quality‐filtered using fastp, and low‐quality reads were removed to obtain clean reads. Gene expression levels were quantified as fragments per kilobase of transcript per million mapped reads (FPKM), and read counts were obtained using HTSeq‐count. PCA was performed in R to evaluate biological replicates. Differential gene expression analysis was conducted using the “DESeq2” package, with a q‐value cutoff of 0.05 and |log2FC| > 1.5. Enrichment analysis was performed using the “clusterProfiler” package based on the hypergeometric distribution to identify significantly enriched GO terms and KEGG pathways.

### Marrow Transplantation

Recipient mice underwent macrophage depletion via intravenous administration of 200 µL clodronate liposomes. Two days later, BMDMs from wild‐type (WT) or Ccr1‐knockout (Ccr1^‐KO^) mice, labeled with PKH26, were intravenously injected into recipient mice. Simultaneously, the CLP model was established.^[^ ^59^ ^]^

### Seahorse XFe Metabolic Flux Analysis

Extracellular acidification rate (ECAR) and oxygen consumption rate (OCR) were measured using an XFe96 Extracellular Flux Analyzer. Macrophages were seeded at a density of 5×10^4 cells/well in 96‐well plates and allowed to adhere overnight. The following day, cells were incubated with XF assay buffer and placed in a CO_2_‐free incubator for 1 h. Glucose, oligomycin, and 2‐deoxy‐glucose (2‐DG) were automatically injected to determine ECAR, while oligomycin, FCCP, and rotenone/antimycin A (Rot/AA) were used to measure OCR. All procedures were performed according to the manufacturer's instructions.

### ChIP‐qPCR

ChIP‐qPCR analysis of BMDMs was conducted to investigate the potential of STAT1 to directly modulate glycolysis. Cells were harvested, minced, rinsed, crosslinked with formaldehyde, quenched with glycine, disrupted, and lysed. Nuclei were isolated, and DNA was digested and sheared into 150–250 bp fragments. A 2% aliquot served as input control, and the remainder was immunoprecipitated with anti‐STAT1 antibody or IgG overnight at 4°C. The next day, complex washing, chromatin elution, reverse crosslinking with proteinase K, and DNA purification were sequentially performed. ChIP‐DNA and input control were analyzed by qPCR using qTower^3^touch (AnalytikJena, Germany).

### Western Blot

Cells were homogenized in RIPA buffer supplemented with protease and phosphatase inhibitor cocktails. Protein extracts were separated on 10% acrylamide gels, transferred onto PVDF membranes, and blotted with primary antibodies. Protein bands were visualized using the Enhanced ECL Substrate Kit.

### Real‐Time Reverse Transcription‐Quantitative Polymerase Chain Reaction (RT‒qPCR)

Total RNA was extracted using TRIzol Reagent and reverse transcribed into cDNA. qPCR was performed using ChamQ Universal SYBR qPCR Master Mix on the qTower^3^touch (AnalytikJena, Germany). Relative mRNA expression was normalized to β‐actin and reported as fold change.

### Coimmunoprecipitation (Co‐IP)

Cells were lysed with NP‐40 lysis buffer. The harvested cell lysates were incubated overnight with primary antibody and protein A/G agarose beads at 4°C. The protein mixture was washed with NP‐40 lysis buffer 3 times, centrifuged at 1500 rpm for 5 min at 4°C, and detected by western blot.

### IHC Staining

Samples were dewaxed with xylene, rehydrated with graded ethanol solutions, and subjected to antigen retrieval. Sections were incubated with 3% hydrogen peroxide for 15 min to block endogenous peroxidase activity and 5% goat serum for 1 h to prevent nonspecific staining. Sections were incubated with primary antibodies overnight at 4°C, followed by secondary antibodies and DAB staining buffer. Quantitative analysis was performed using ImageJ software.

### Immunofluorescence

Cells or slides were then fixed with 4% paraformaldehyde for 10 min, permeabilized with 0.2% Triton X‐100 for 20 min, blocked with 5% BSA for 1 h at room temperature, and stained with antibodies overnight at 4 °C. Secondary antibodies conjugated with specific fluorescent dyes were applied for 2 h at room temperature, followed by DAPI staining. Samples were imaged using a confocal laser scanning microscope (Olympus, Japan), and 6 random fields were counted using ImageJ software.

### Migration and Invasion Assays

Transwell chambers with or without a Matrigel coating were prepared in 24‐well plates. Subsequently, 1×10^5 BMDMs were seeded in the serum‐free medium in the upper chamber, and 500 µl of RPMI 1640 medium containing 20% fetal bovine serum was added to the lower chamber. Cells were incubated at 37 °C for 24 (migration) or 48 h (invasion). Non‐migrated/invasive cells were removed with a cotton swab, and migrated/invasive cells were fixed with cold methanol and stained with 0.1% crystal violet. Images were captured under a microscope, and cell numbers were counted.

### Evans Blue Staining

Lung permeability was measured by injecting 2% Evans blue dye (4 mL kg^−1^) via the tail vein. After 1 h, mice were anesthetized, and the right ventricle was perfused with 20 mL of normal saline for 2 min. Lung tissues were excised, weighed, and homogenized with formamide (1 mL/100 mg) at 55°C for 24 h. Following centrifugation at 12000 rpm for 20 min, the concentration of Evans blue in the supernatant was determined spectrophotometrically at 620 nm.

### Terminal Deoxynucleotidyl Transferase dUTP Nick End Labeling (TUNEL) Assay

Paraffin‐embedded intestinal tissue sections were subjected to TUNEL assays to identify apoptotic cells using the TUNEL BrightRed Apoptosis Detection Kit according to the manufacturer's instructions. Sections were co‐stained with an anti‐CD31 antibody to identify endothelial cells, and nuclei were counterstained with DAPI.

### HE Staining and Masson's Trichrome Staining

Lung samples were fixed in buffered formalin, embedded in paraffin, and sectioned at 4 µm. Sections were deparaffinized, rehydrated, and stained with hematoxylin for 5 min and eosin for 2 min. Lung lesions were evaluated using a modified Masson's trichrome staining kit according to the manufacturer's guidelines. Stained sections were imaged using a microscope (Olympus, Japan), and lung injury scores were calculated based on a previously described method.^[^ ^60^ ^]^

### Statistical Analysis

Data are presented as the mean ± standard deviation (SD) from at least 3 independent experiments. P value <0.05 was considered to indicate statistical significance. All the statistical analyses were performed via GraphPad Prism software. For data from two groups, unpaired or paired Student's t‐tests were conducted. One‐way ANOVA followed by Tukey's post hoc test or Scheffe's post hoc test was performed to compare the significance of differences among more than 2 groups. Two‐way ANOVA followed by Tukey's post hoc test or Scheffe's post hoc test was used for multiparameter analyses.

## Conflict of Interest

The authors declare no conflicts of interest.

## Author Contributions

X.L., Y.Q.L., and Y.X.Z. contributed equally to this work. C.H.M. conceptualized the study and was responsible for funding acquisition, resources, and supervision. X.L. performed the experiments and drafted the manuscript. X.L., Y.Q.L., and Y.X.Z. were responsible for the methodology. X.L., Y.Q.L., and Y.X.Z. were responsible for the investigation. J.H.G. and P.Z. were responsible for sample collection.

## Supporting information



Supporting Information

## Data Availability

The data that support the findings of this study are available from the corresponding author upon reasonable request.
