# Supplementary material for: Endothelial‐Derived CCL7 Promotes Macrophage Polarization and Aggravates Septic Acute Lung Injury via CCR1‐Mediated STAT1 Succinylation
Source: Adv Sci (Weinh). 2025 Aug 4;12(38):e06209. doi: 10.1002/advs.202506209 (PMC12520477; doi:10.1002/advs.202506209)
Supplement: Supplementary file 1 — Supporting Information [file ADVS-12-e06209-s001.pdf]

ADVANCED SCIENCE
Open Access

Supporting Information

for *Adv. Sci.*, DOI 10.1002/advs.202506209

Endothelial-Derived CCL7 Promotes Macrophage Polarization and Aggravates Septic Acute Lung Injury via CCR1-Mediated STAT1 Succinylation

*Xue Li, Yuqin Long, Yunxi Zhu, Jiahui Gu, Ping Zhou and Changhong Miao**

Supplementary Materials for

Endothelial-derived CCL7 promotes macrophage polarization and aggravates septic ALI via CCR1-mediated STAT1 succinylation

Xue Li[1,2], Yuqin Long[1,2], Yunxi Zhu[1,2], Jiahui Gu[1,2], Ping Zhou[1,2], Changhong Miao [1,2*]

1. Department of Anesthesiology, Zhongshan Hospital, Fudan University, Shanghai, China

2. Shanghai Key Laboratory of Perioperative Stress and Protection, Shanghai, China

[*]Corresponding author. Department of Anesthesiology, Zhongshan Hospital, Fudan University, 180# Feng-Lin Road, Shanghai, China.

E-mail addresses: miaochh @aliyun.com.

This PDF file includes:

Supplementary Figures 1 to 10

Supplementary Table 1-2

Supplementary Figures:

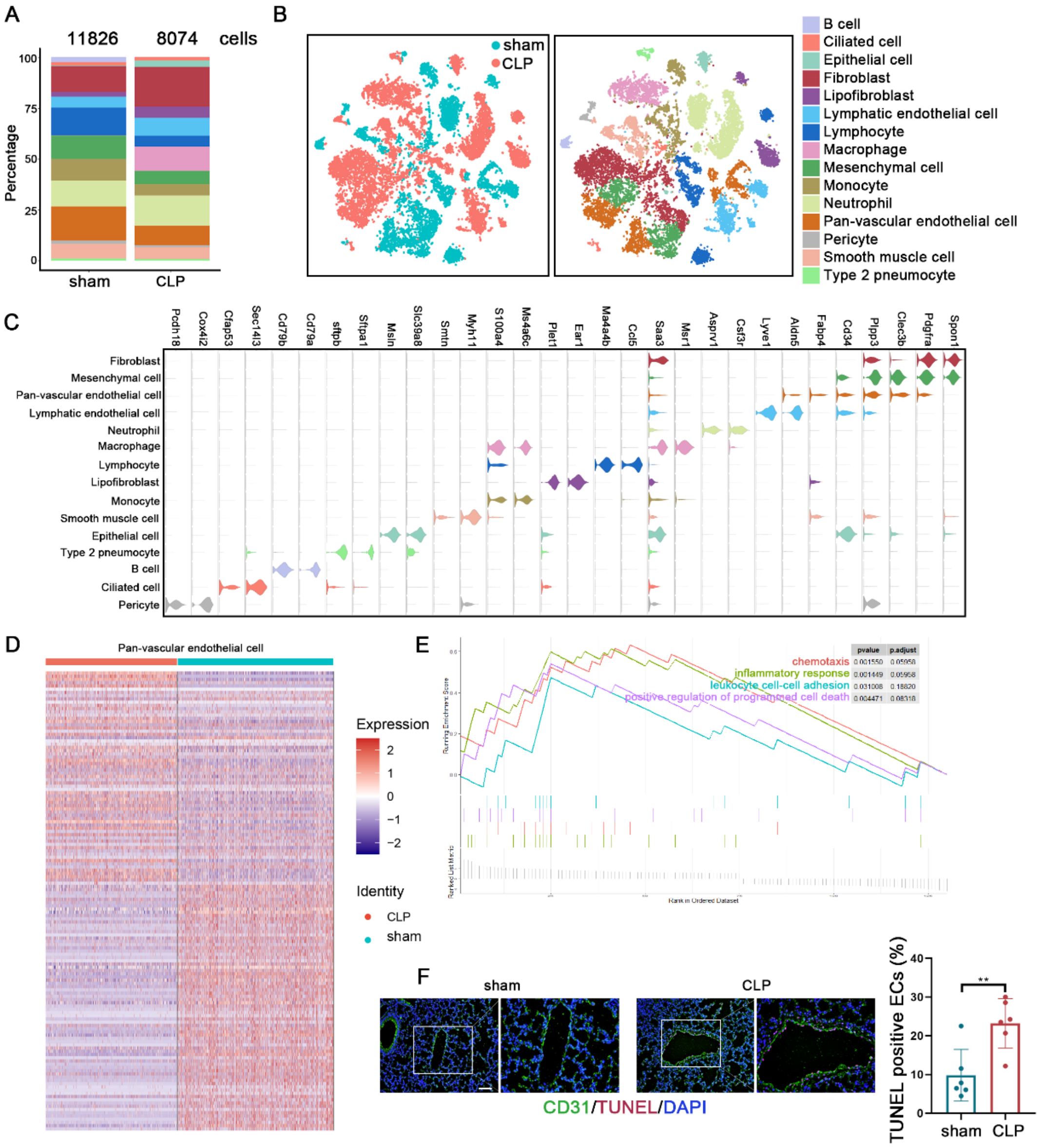

Supplementary Figure1. Inhibition of endothelial-derived CCL7 improves septic ALI.

A) Overlay histogram illustrating the total number of mouse lung tissue cells, and the proportion of each cell type derived from single-cell sequencing analysis of the sham and CLP groups. B) Combined t-SNE plot displaying 15 clusters of the sham and CLP groups (left) and subclusters of lung cells (right). C) Violin plots depicting marker gene expression levels for cell subpopulation annotation. D) Heatmap representing DEGs identified from single-cell sequencing data of pan-vascular endothelial cells between the sham and CLP mice. E) GSEA showing significant response pathways of

pan-vascular endothelial cells in the CLP mice. F) Representative images of TUNEL (red) and CD31 (green) co-staining in the lung sections of the sham and CLP mice, with a histogram indicating the percentage of $TUNEL^{+}CD31^{+}$ cells among the ECs (scale bar:100μm, n = 6). Data are presented as mean ± SD, $^{*}p < 0.05$, $^{**}p < 0.01$. Data in F were analyzed by two-tailed Student's t-test.

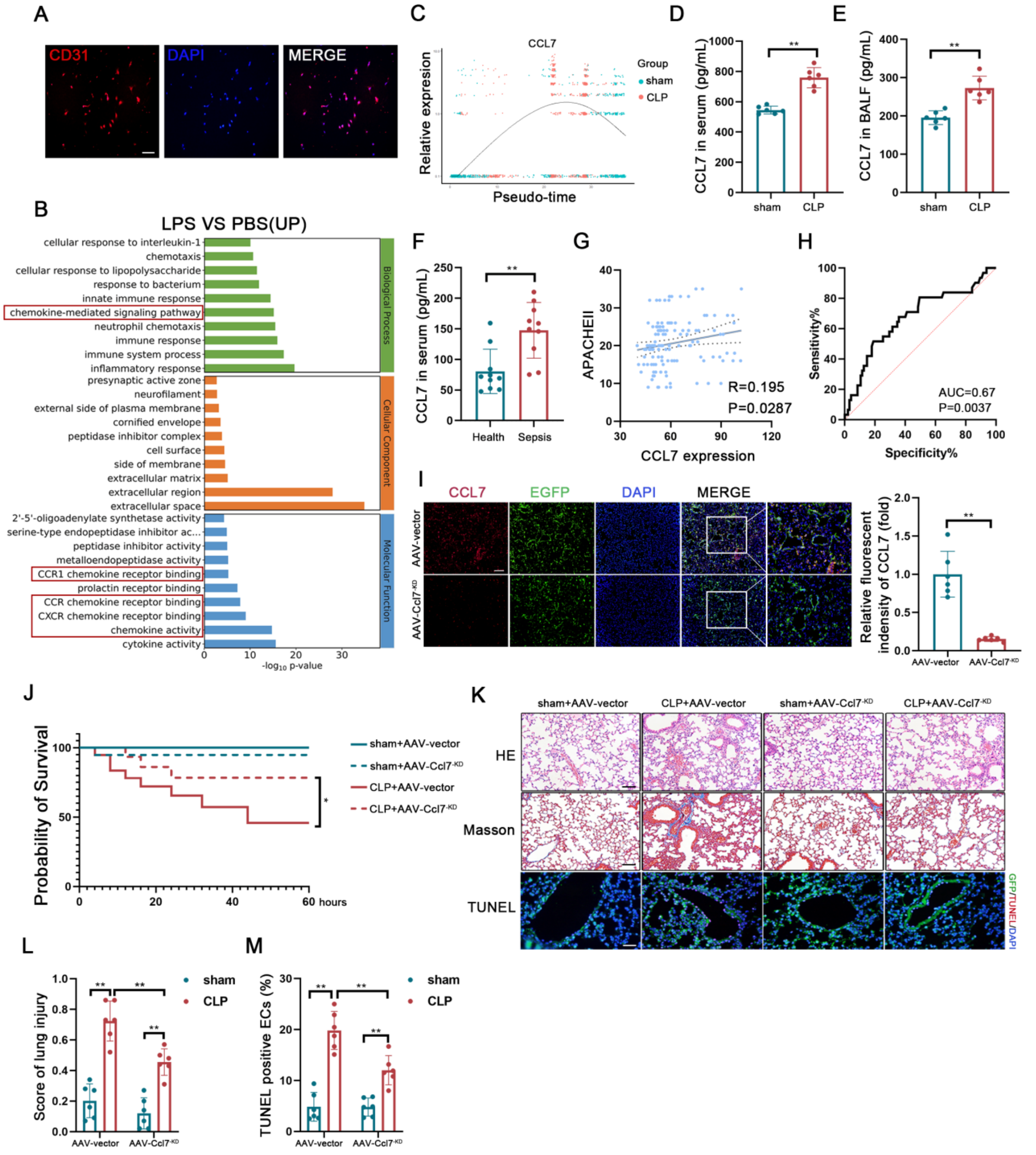

Supplementary Figure 2. Inhibition of endothelial-derived CCL7 improves septic ALI.

A) Immunofluorescence staining confirmed the specificity of $CD31^{+}$ (red) ECs (scale bar: 100μm). B) GO enrichment analysis of the top-upregulated 30 terms in the ECs treated with LPS for 24h. C) Pseudo-time trajectory analysis of CCL7 expression changes in pan-vascular endothelial cells in the sham and CLP groups. D, E) CCL7 concentrations in the serum (D) and BALF (E) of the sham and CLP mice (n = 6). F) CCL7 concentration in the serum of healthy volunteers and sepsis patients (n = 10). G) Pearson correlation matrix between CCL7 expression levels and the APACHEII

scores of septic patients. H) ROC curve based on a prediction model distinguishing high and low CCL7 expression in septic patients. I) Representative immunofluorescence images verifying the knockdown efficiency of AAV-Ccl7^{-KD} in the mouse lung tissue (scale bar: 100μm, n = 6). J) Kaplan-Meier survival curve comparing AAV-vector and AAV-Ccl7^{-KD} mice with or without sepsis (n = 10). K-M) Representative HE staining (upper, scale bar: 100μm), Masson trichrome staining (middle, scale bar: 100μm), and TUNEL staining (lower, scale bar: 50μm) of lung sections from AAV-vector and AAV-Ccl7^{-KD} mice with or without sepsis. The lung injury scores were evaluated in a blinded manner, and the histogram showed the percentage of TUNEL^{+}CD31^{+} cells among the ECs (n = 6). Data are presented as mean ± SD, *$p < 0.05$, **$p < 0.01$. Data in D-F, and I were analyzed by two-tailed Student's t-test. Data in J was analyzed by the Log-rank test. Data in L and M were analyzed by two-way ANOVA with Tukey's post hoc test.

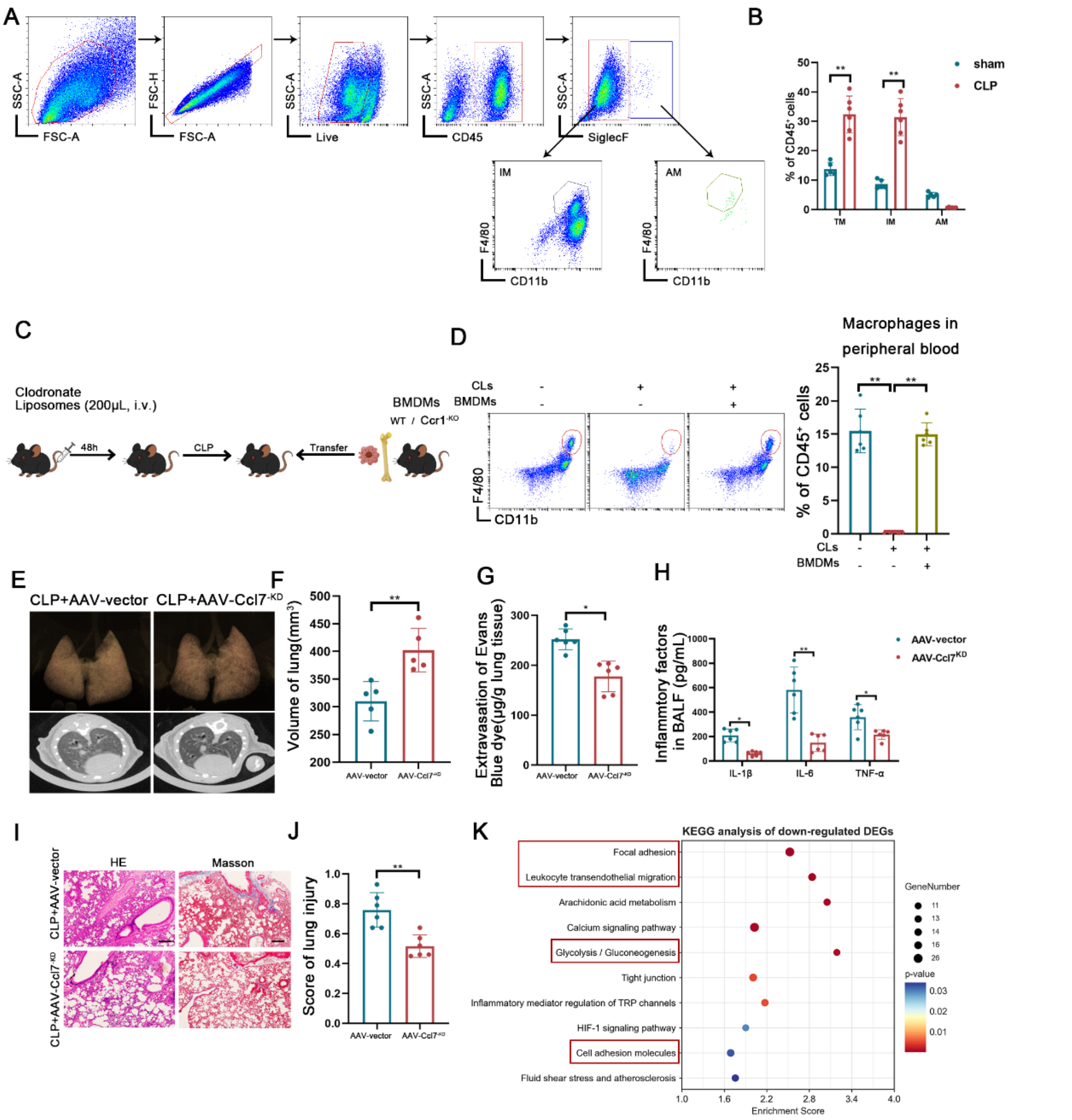

Supplementary Figure 3. CCL7 regulates infiltration and inflammation of CCR1$^+$ macrophages.

A) Representative gating strategy for sorting Live$^+$CD45$^+$SiglecF$^-$CD11b$^+$F4/80$^+$ IMs and Live$^+$CD45$^+$SiglecF$^+$CD11b$^-$F4/80$^+$ AMs. B) Proportions of the IMs, AMs, and TMs in the lung tissue of sham and CLP mice (n = 5/6). C) Schematic diagram illustrating the experimental design for constructing chimera models. D) Flow cytometry showing the efficiency of macrophage depletion and adoptive transfer in the peripheral blood of mice (n = 5/6). E) 3D imaging and CT scans of the lung from AAV-vector and AAV-Ccl7^{-KD} septic mice transfused with BMDMs. F) Quantification of micro-CT-derived non-aerated lung volume as an indicator of lung consolidation in

AAV-vector and AAV-Ccl7^{-KD} septic mice transfused with BMDMs (n = 5). G) Assessment of lung transvascular permeability by measuring Evans blue dye leakage in micrograms per gram of lung tissue in AAV-vector and AAV-Ccl7^{-KD} septic mice transfused with BMDMs (n = 6). H) Concentrations of IL-1β, IL-6, and TNF-α in the BALF of AAV-vector and AAV-Ccl7^{-KD} septic mice transfused with BMDMs (n = 6). I, J) Representative HE staining (upper, scale bar: 100μm), and Masson trichrome staining (lower, scale bar: 100μm) of lung sections from AAV-vector and AAV-Ccl7^{-KD} septic mice. The lung injury scores were evaluated in a blinded manner (n = 6). K) KEGG analysis of top-upregulated 10 terms in PKH26-labeled BMDMs from AAV-vector and AAV-Ccl7^{KD} septic mice. Data are presented as mean ± SD, $*p < 0.05$, $**p < 0.01$. Data in B, F-H, and J were analyzed by two-tailed Student's t-test. Data in D were analyzed by two-way ANOVA with Tukey's post hoc test.

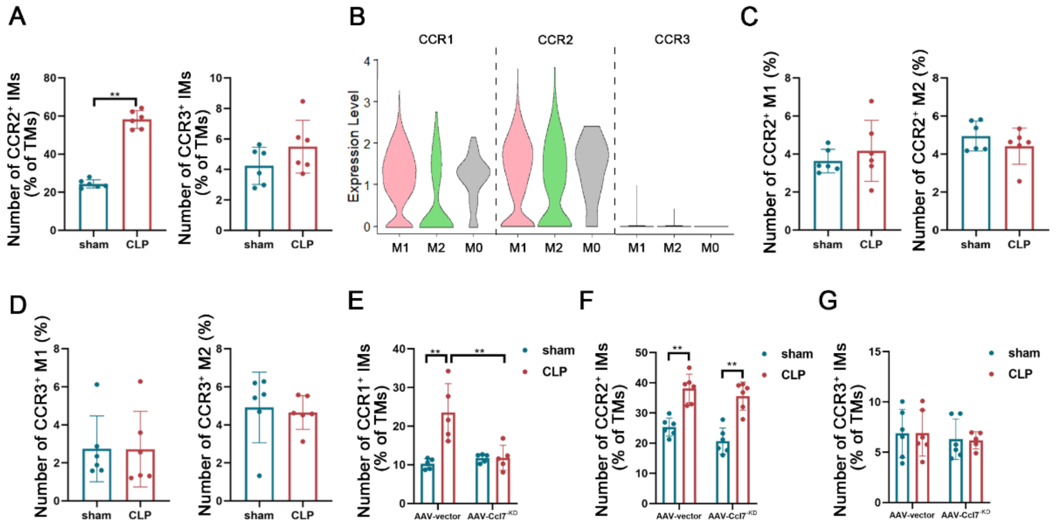

Supplementary Figure 4. CCL7 regulates infiltration and inflammation of CCR1$^+$ macrophages.

A) Proportions of CCR2$^+$ IMs or CCR3$^+$ IMs among the TMs in the lung tissue of sham and CLP mice (n = 6). B) Violin plots depicting normalized expression of CCR1, CCR2, and CCR3 across macrophage clusters. C) Proportions of CD86$^+$ M1 macrophages or CD206$^+$ M2 macrophages among the CCR2$^+$ IMs in the lung tissue of sham and CLP mice (n = 6). D) Proportions of CD86$^+$ M1 macrophages or CD206$^+$ M2 macrophages among the CCR3$^+$ IMs in the lung tissue of sham and CLP mice (n = 6). E) Proportions of the CCR1$^+$ IMs in the lung tissue of the AAV-vector and AAV-Ccl7^{-KD} mice with or without sepsis (n = 5). F) Proportions of the CCR2$^+$ IMs in the lung tissue of the AAV-vector and AAV-Ccl7^{-KD} mice with or without sepsis (n = 5). G) Proportions of the CCR3$^+$ IMs in the lung tissue of the AAV-vector and AAV-Ccl7^{-KD} mice with or without sepsis (n = 5). Data are presented as mean ± SD, *$p < 0.05$, **$p < 0.01$. Data in A, C, and D were analyzed by two-tailed Student's t-test. Data in E and F were analyzed by two-way ANOVA with Tukey's post hoc test. Data in G were analyzed by two-way ANOVA with Scheffe's post hoc test.

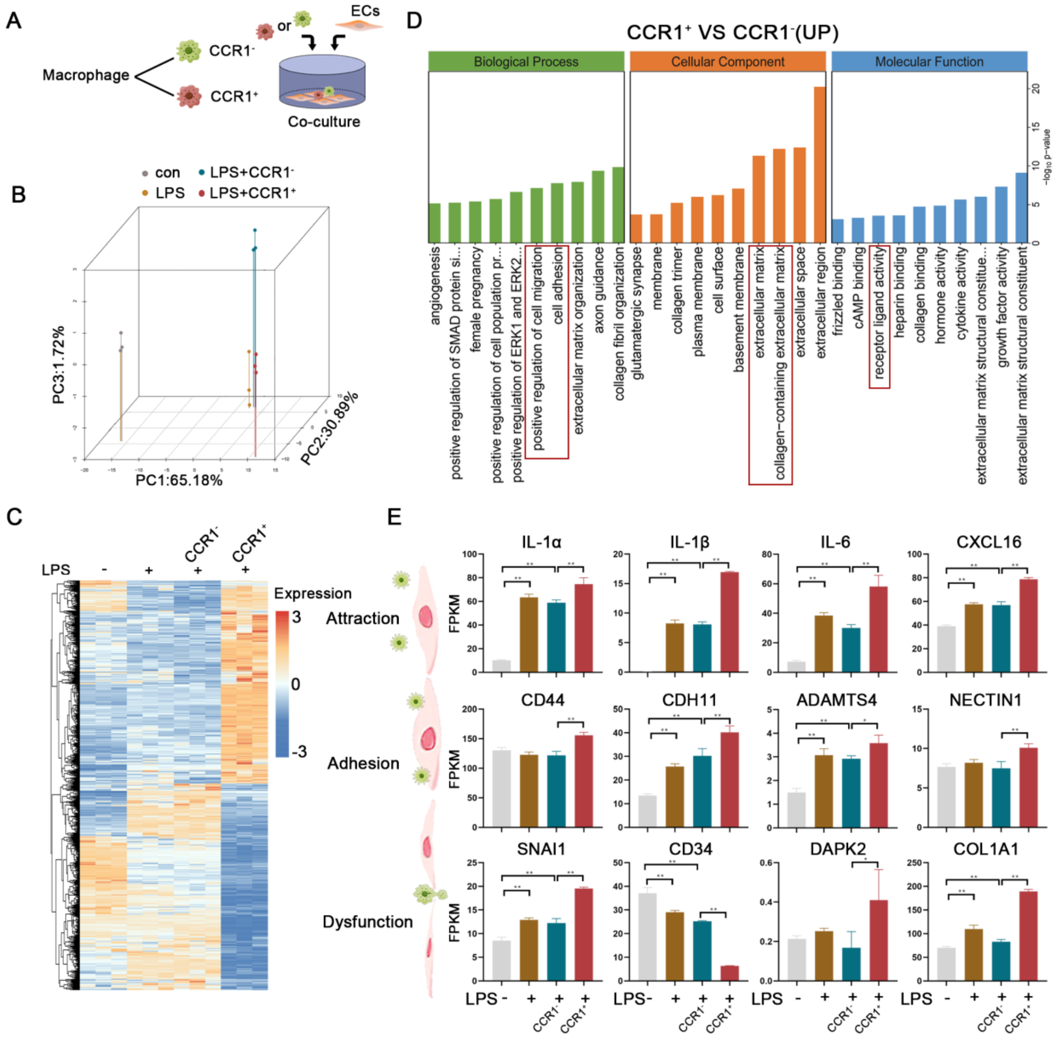

Supplementary Figure 5. CCL7 regulates infiltration and inflammation of CCR1^{+} macrophage.

A) ECs were co-cultured with CCR1^{-} BMDMs or CCR1^{+} BMDMs for 24h. B) Three-dimensional principal component analysis (3D-PCA) visualization depicting the distribution of samples in a space composed of three principal components (PC1=65.18%, PC2=30.89%, PC3=1.72%). C) Heatmap representing DEGs derived from RNA sequencing data of LPS-treated ECs co-cultured with CCR1^{-} BMDMs or CCR1^{+} BMDMs. D) GO enrichment analysis of the top-upregulated 30 terms in ECs co-cultured with CCR1^{-} BMDMs or CCR1^{+} BMDMs. E) Regulation of mediators of attraction (IL-1α, IL-1β, IL-6 and CXCL16), adhesion markers (CD44, CDH11, ADAMTS4 and NECTIN1), and dysfunction markers (CD34, SNAI1, DAPK2, and COL1A1) in ECs co-cultured with CCR1^{-} BMDMs or CCR1^{+} BMDMs (n = 3). Data

in E were analyzed by one-way ANOVA with Tukey's post hoc test.

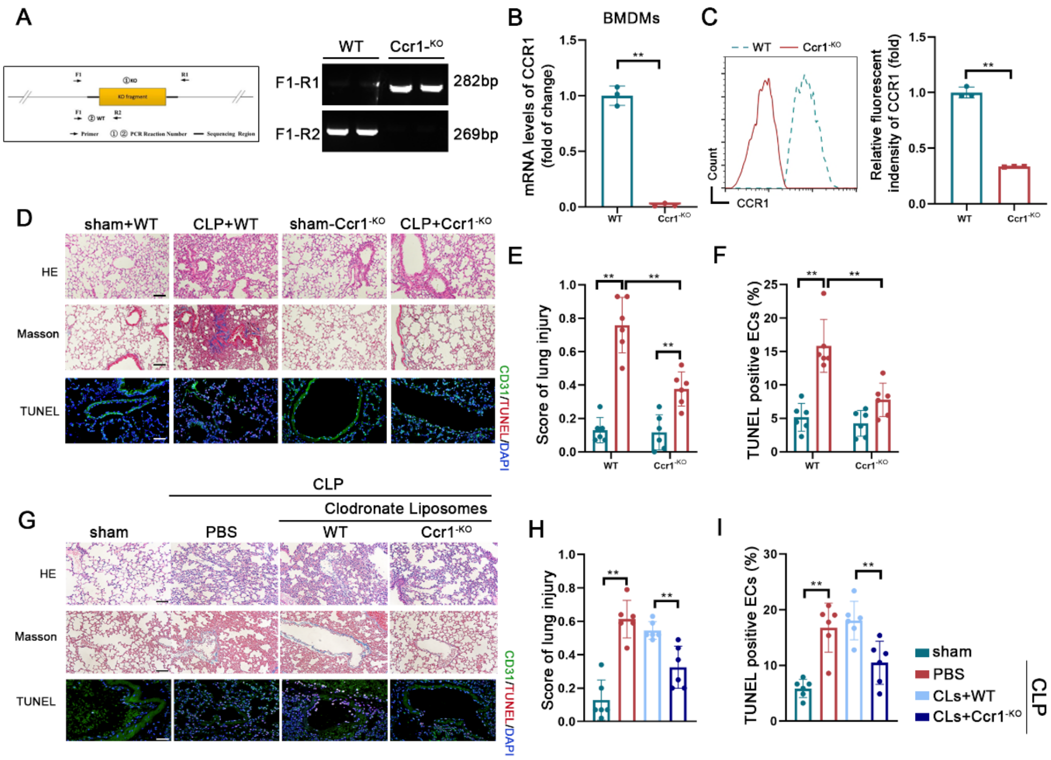

Supplementary Figure 6. CCR1$^+$ macrophages play a crucial role in septic ALI.

A) Schematic diagram of the transgenic construct architecture and PCR primer localization. Agarose gel analysis of PCR genotyping identifies wild-type(WT) and homozygous Ccr1-knockout (Ccr1^{-KO}) transgenic mice. B) Relative mRNA expression of CCR1 in the BMDMs from WT and Ccr1^{-KO} mice (n = 3). C) Relative fluorescent indensity of CCR1 in the BMDMs from WT and Ccr1^{-KO} mice (n = 3). D-F) Representative HE staining (upper, scale bar: 100μm), Masson trichrome staining (middle, scale bar: 100μm), and TUNEL staining (lower, scale bar: 50μm) of lung sections from WT and Ccr1^{-KO} mice with or without sepsis. The lung injury scores were evaluated in a blinded manner, and the histogram showed the percentage of TUNEL$^+$CD31$^+$ cells among ECs (n = 6). G-I) Representative HE staining (upper, scale bar: 100μm), Masson trichrome staining (middle, scale bar: 100μm), and TUNEL staining (lower, scale bar: 50μm) of lung sections from the indicated groups. The lung injury scores were evaluated in a blinded manner, and the histogram showed the percentage of TUNEL$^+$CD31$^+$ cells among ECs (n = 6). Data are presented as mean ± SD, *$p < 0.05$, **$p < 0.01$. Data in B and C were analyzed by two-tailed

Student's t-test. Data in E and F were analyzed by two-way ANOVA with Tukey's post hoc test. Data in H and I were analyzed by one-way ANOVA with Tukey's post hoc test.

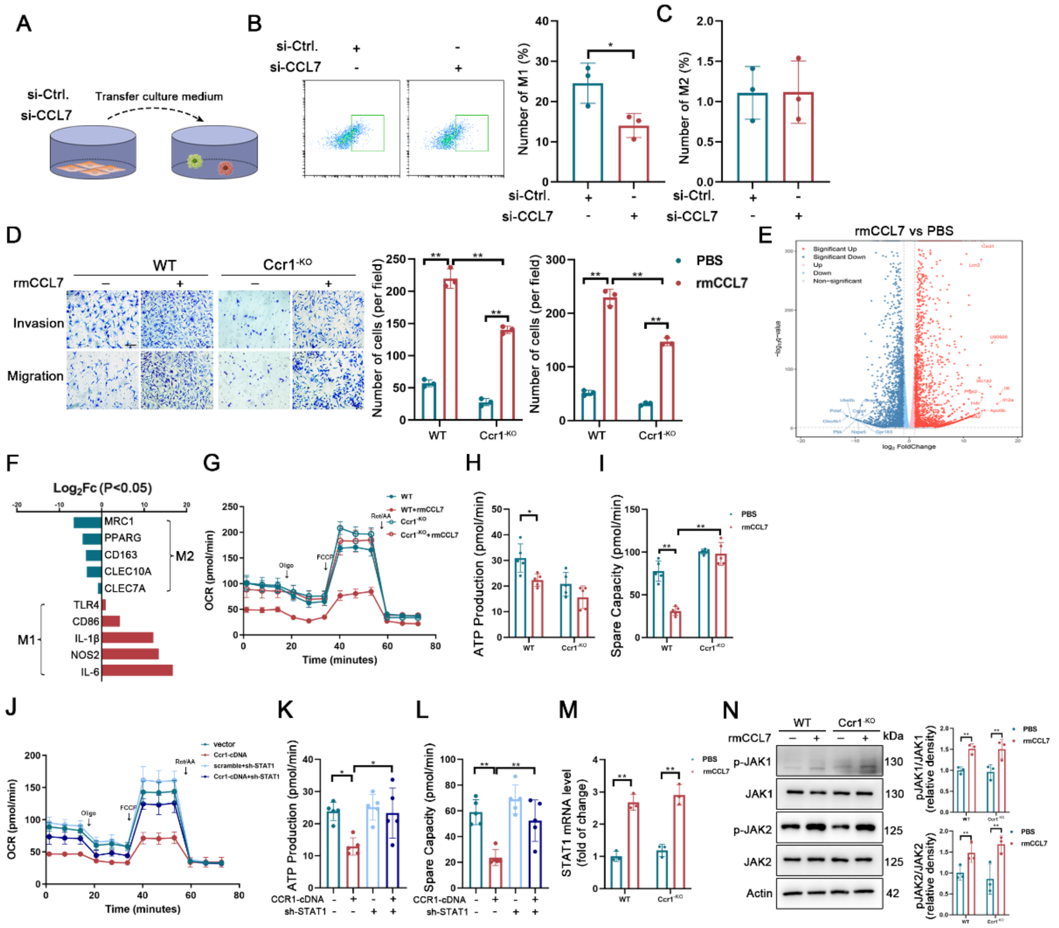

Supplementary Figure 7. The CCL7–CCR1 axis regulates macrophage polarization via STAT1 activation.

A) Culture medium from the si-Ctrl. and si-CCL7 ECs was transferred to BMDMs for 24 h. B, C) Proportions of $CD86^+$ M1 macrophages (B) or $CD206^+$ M2 macrophages (C) among the BMDMs in the indicated groups (n = 3). D) Invasion and migration abilities of WT and $Ccr1^{-KO}$ BMDMs with or without rmCCL7 (scale bar: 100μm, n = 3). E) Volcano plot depicting the DEGs identified by RNA sequencing analysis. F) Log_2FC value of marker genes of M1 (TLR4, CD86, IL-1β, NOS2, IL-6) and M2 (MRC1, PPARG, CD163, CLEC10A, CLEC7A) macrophages. G-I) OCR in WT and $Ccr1^{-KO}$ BMDMs with or without rmCCL7 (n = 5). J-L) OCR in the indicated groups (n = 5). M) Relative mRNA levels of STAT1 in WT and $Ccr1^{-KO}$ BMDMs treated with or without rmCCL7 (n = 3). N) Western blot analysis of JAK1/2 and p-JAK1/2 expression in WT and $Ccr1^{-KO}$ BMDMs treated with or without rmCCL7 (n = 3). Data are presented as mean ± SD, ns, nonsignificant, *p < 0.05, **p < 0.01. Data in B and

C were analyzed by two-tailed Student's t-test. Data in D, H, I, M, and N were analyzed by two-way ANOVA with Tukey's post hoc test. Data in K and L were analyzed by one-way ANOVA with Tukey's post hoc test.

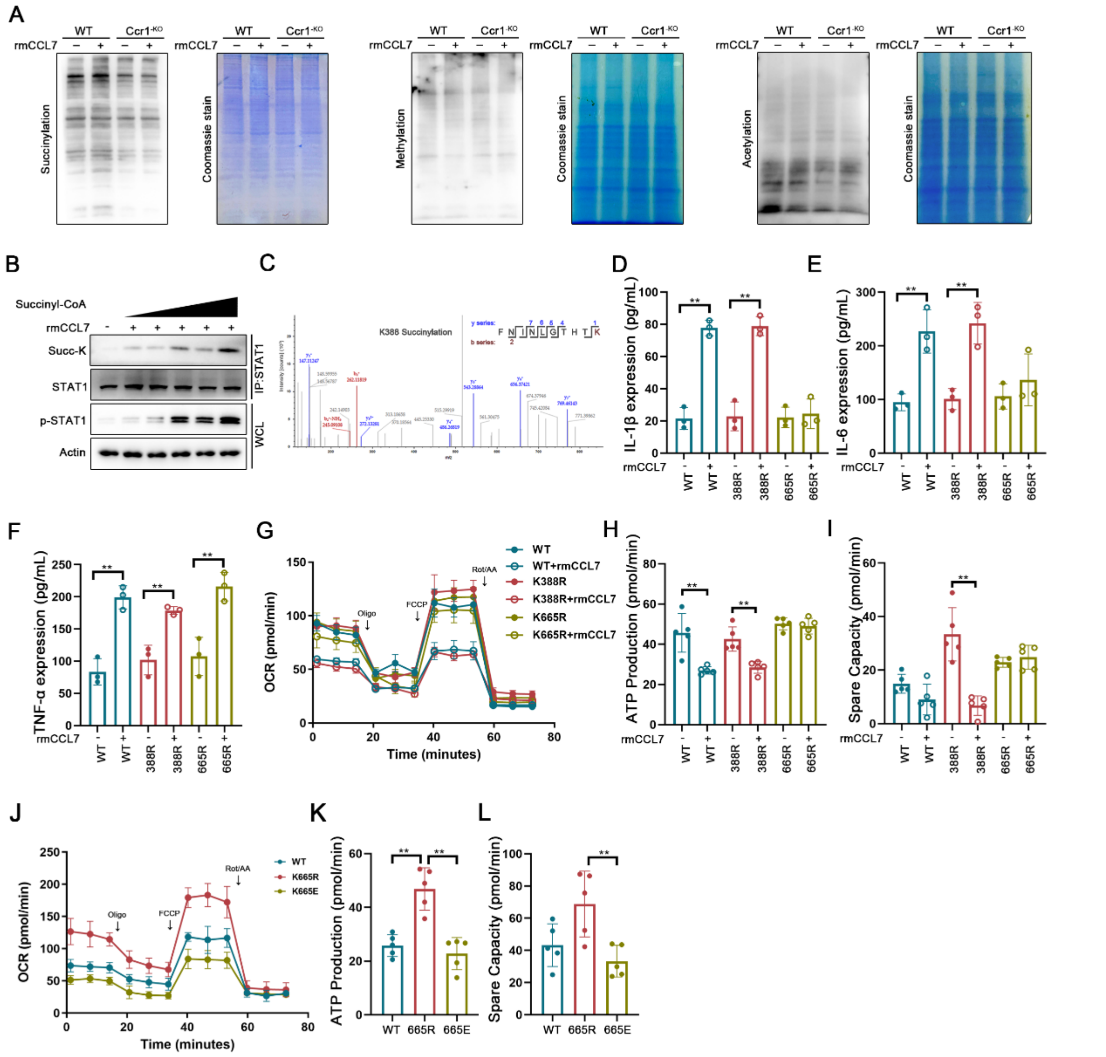

Supplementary Figure 8. Metabolic reprogramming in macrophages is dependent on STAT1^{-K665} succinylation.

A) Western blot analysis of succinylation, methylation, and acetylation levels in WT and Ccr1^{-KO} BMDMs treated with or without rmCCL7. B) Immunoprecipitation analysis of STAT1 succinylation levels in BMDMs treated with varying concentrations of succinyl-CoA. C) Lysine succinylation proteomics identifying STAT1^{-K388} modification site via LC-MS/MS. D-F) Concentrations of IL-1β, IL-6, and TNF-α in the supernatant of STAT1^{-WT}, STAT1^{-K388R}, and STAT1^{-K665R} BMDMs with or without rmCCL7 (n = 3). G-I) OCR in the STAT1^{-WT}, STAT1^{-K388R}, and STAT1^{-K665R} BMDMs with or without rmCCL7 (n = 5). J-L) OCR in the STAT1^{-WT}, STAT1^{-K665R}, and STAT1^{-K665E} BMDMs (n = 5). Data are presented as mean ± SD, ns, nonsignificant, $^{*}p < 0.05$, $^{**}p < 0.01$. Data in D-F, H, and I were analyzed by

two-tailed Student's t-test. Data in K and L were analyzed by one-way ANOVA with Tukey's post hoc test.

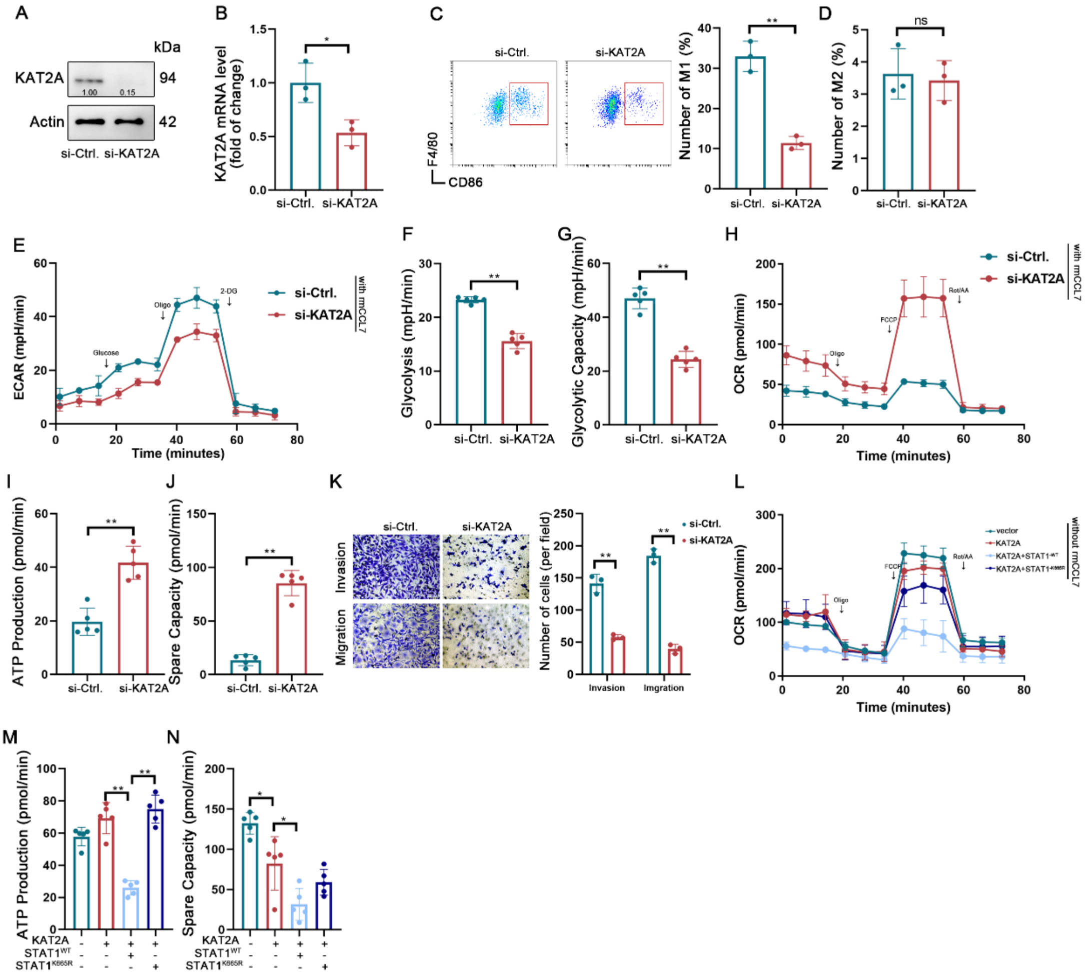

Supplementary Figure 9. The CCL7–CCR1 axis upregulates KAT2A to drive STAT1 succinylation.

A) Western blot analysis of KAT2A protein levels in the indicated groups. B) Relative mRNA expression levels of KAT2A in the indicated groups (n = 3). C, D) Proportion of the CD86^{+} M1 macrophages (C) or CD206^{+} M2 macrophages (D) among the BMDMs in the indicated groups (n = 3). E-G) ECAR in the indicated groups (n = 5). H-J) OCR in the indicated groups (n = 5). K) Invasion and migration abilities of BMDMs in the indicated groups (scale bar: 100μm, n = 3). L-N) OCR in the indicated groups (n = 5). Data are presented as mean ± SD, ns, nonsignificant, *p < 0.05, **p < 0.01. Data in B-D, F, G, and I-K were analyzed by two-tailed Student's t-test. Data in M and N were analyzed by one-way ANOVA with Tukey's post hoc test.

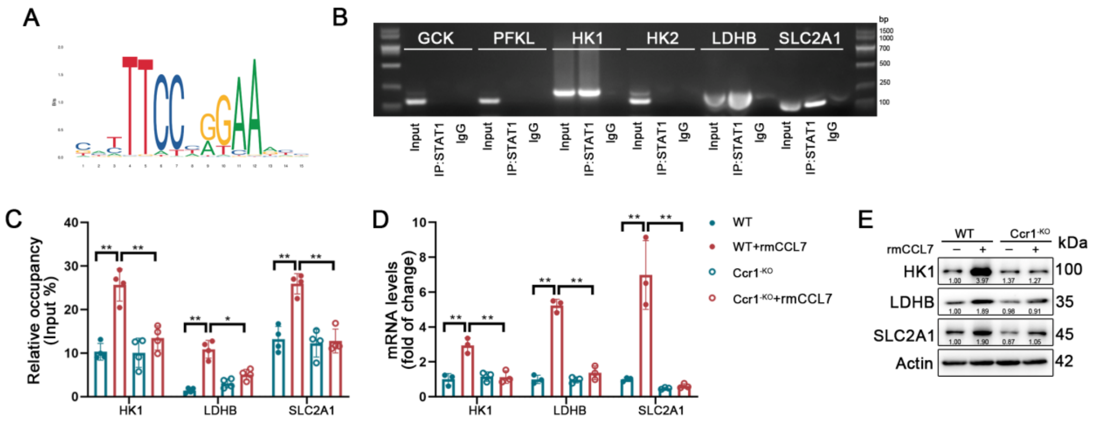

Supplementary Figure 10. The binding of STAT1 to the promoter region of glycolytic genes is increased by succinylation.

A) Predicted binding sequence of the transcription factor STAT1 at promoter regions, as identified by the JASPAR database (http://jaspar.genereg.net). B) Validation of STAT1 occupancy at promoter regions of glycolysis-related genes using ChIP-PCR. C) Quantification of STAT1 peak abundance at the promoters of glycolysis-related genes (HK1, LDHB, SLC2A1) in the indicated groups (n = 3). D) Relative mRNA expression levels of HK1, LDHB, and SLC2A1 in the indicated groups (n = 3). E. Western blot analysis of HK1, LDHB, and SLC2A1 protein levels in the indicated groups. Data are presented as mean ± SD, *p < 0.05, **p < 0.01. Data D was analyzed by two-way ANOVA with Tukey's post hoc test.

Supplementary Table 1		
**Hydrogen bonds**		
**Structure-STAT1**	**Dist. [Å]**	**Structure-KAT2A**
A:LYS 652[N]	2.35	C:GLN 15[OE1]
A:VAL 709[N]	3.76	C:GLN 15[OE1]
A:ASN 391[ND2]	3.80	C:SER 165[OG]
A:LYS 566[NZ]	2.58	C:PRO 225[O]
A:LYS 567[NZ]	3.20	C:LYS 228[O]
A:ASN 460[ND2]	3.57	C:GLN 233[O]
A:ASN 460[ND2]	2.48	C:ASN 237[OD1]
A:LYS 636[NZ]	3.66	C:TRP 375[O]
A:LYS 636[NZ]	3.37	C:GLU 376[OE1]
A:ARG 608[NH2]	3.23	C:GLY 378[O]
A:SER 462[OG]	3.84	C:PHE 379[O]
A:ARG 683[NH1]	3.73	C:PRO 475[O]
A:GLN 95[NE2]	3.07	C:GLU 488[OE1]
A:ARG 88[NE]	3.60	C:THR 489[OG1]
A:SER 69[OG]	2.75	C:ASP 556[OD2]
A:ARG 84[NH1]	3.23	C:ASN 584[O]
A:ARG 84[NH2]	2.79	C:GLU 585[O]
A:TYR 68[OH]	2.35	C:GLU 585[OE1]
A:ARG 84[NH1]	3.05	C:GLU 585[OE2]
A:ARG 88[NH2]	3.46	C:VAL 587[O]
A:ASP 65[OD2]	3.80	C:ARG 496[NH1]
A:TYR 68[OH]	2.69	C:LYS 588[NZ]
A:ASN 93[O]	2.90	C:THR 477[OG1]
A:ASP 97[OD2]	3.75	C:GLY 474[N]
A:GLN 340[OE1]	3.86	C:ARG 310[NH2]
A:TYR 356[OH]	2.96	C:GLU 166[N]
A:TYR 356[OH]	3.39	C:ASP 167[N]
A:MET 392[O]	2.72	C:SER 165[OG]
A:GLU 393[OE1]	3.33	C:ARG 214[NH2]
A:GLU 403[OE2]	2.23	C:ARG 214[NH1]
A:SER 462[OG]	3.34	C:GLN 233[NE2]
A:GLU 559[OE2]	3.85	C:SER 377[OG]
A:GLU 563[OE1]	2.60	C:LYS 228[N]
A:SER 640[OG]	3.38	C:ARG 323[NH1]
A:ILE 647[O]	3.10	C:GLN 15[NE2]
A:GLU 686[OE1]	2.21	C:LYS 676[NZ]

Supplementary Table 2		
**REAGENT or RESOURCE**	**SOURCE**	**IDENTIFIER**
**Antibodies**		
TruStain FcX™ PLUS (anti-mouse CD16/32) Antibody	BioLegend	Cat.156603
Zombie Yellow™ Fixable Viability Kit	BioLegend	Cat.423104
Brilliant Violet 421™ anti-mouse CD45	BioLegend	Cat. 147719
Alexa Fluor® 700 anti-mouse CD45	BioLegend	Cat. 103128
Brilliant Violet 421™ anti-mouse CD170 (Siglec-F)	BioLegend	Cat. 155509
PerCP anti-mouse/human CD11b	BioLegend	Cat. 101230
PE anti-mouse F4/80	BioLegend	Cat. 111604
PE/Cyanine7 anti-mouse CD206	BioLegend	Cat. 141720
APC Rat anti-Mouse CD86	BD Biosciences	Cat. 558703
FITC anti-mouse CD191 (CCR1)	BioLegend	Cat.152506
FITC anti-mouse CD192 (CCR2)	BioLegend	Cat.150607
FITC anti-mouse CD193 (CCR3)	BioLegend	Cat.144510
Anti-β-Actin	Proteintech	Cat. 66009-1-Ig
Anti-Lamin A/C	Proteintech	Cat. 10298-1-AP
Anti-STAT1- ChIP Grade	Abcam	Cat.ab234400
Anti-p-STAT1 (Y701)	Abcam	Cat.ab109457
Anti-MCP-3 (CCL7)	Abcam	Cat.ab228979
Anti-CD86	Abcam	Cat.ab317266
Anti-CD31	Abcam	Cat.ab28364
Anti-Succinyllysine Mouse mAb	PTM Biolabs	Cat. PTM-401
Anti-Acetyllysine Mouse mAb	PTM Biolabs	Cat. PTM-102
Anti-Di-Methyllysine Rabbit pAb	PTM Biolabs	Cat. PTM-606
Anti-KAT2A/GCN5	Proteintech	Cat. 66575-1-Ig
Anti-CPT1A	Proteintech	Cat. 15184-1-AP
Anti-SIRT5	CST	Cat. 8782
Anti-SIRT7	CST	Cat. 5360

Anti-JAK1	CST	Cat. 3332
Anti-P-JAK1 (Y1034/1035)	CST	Cat. 3332
Anti-JAK2	CST	Cat. 3230
Anti-P-JAK2 (Y007/1008)	CST	Cat. 3771
Anti-HK1	Proteintech	Cat. 19662-1-AP
Anti-LDHB	Proteintech	Cat. 14824-1-AP
Anti-SLC2A1	Proteintech	Cat. 21829-1-AP
Anti-HA-tag	CST	Cat.3724
Anti-Flag-tag	CST	Cat.14793
Anti-Myc-tag	CST	Cat.13987
Anti-Ubiquitin	CST	Cat.3936
Anti-IgG	Proteintech	Cat. SA00001-2
Alexa Fluor 488-labeled Goat Anti-Rabbit IgG(H+L)	Yeasen	Cat. 33106ES60
Alexa Fluor 594-labeled Goat Anti-Mouse IgG(H+L)	Yeasen	Cat.33212ES60
HRP AffiniPure Goat Anti-Mouse IgG(H+L)	Proteintech	Cat. SA00001-1
HRP AffiniPure Goat Anti-Rabbit IgG(H+L)	Proteintech	Cat. SA00001-2
**Chemicals, peptides, and recombinant proteins**		
CollagenaseIV	Sigma Aldrich	Cat. 9001-12-1
DispaseII	Sigma Aldrich	Cat. D4693
DnaseI	Sigma Aldrich	Cat. 11284932001
Fetal bovine serum(PBS)	Gibco	Cat. 10099-141C
Endothelial cell growth supplement (ECGS)	ScienCell	Cat. 1052
Penicillin-streptomycin solution (P/S)	ScienCell	Cat. 0503
Lipopolysaccharide (LPS)	Sigma Aldrich	Cat.L2630
M-CSF/CSF1 Protein, Mouse, Recombinant	TargetMol	Cat. TMPY-00464
Red blood cell lysis buffer	Solarbio	Cat.R1010-500ml
CD31 microbeads	Miltenyi Biotech	Cat.130-097-418
Corning® Matrigel® Matrix	Corning	Cat.356234
Recombinant Mouse CCL7/MARC Protein	R&D Systems	Cat.456-MC-010

Phosphate Buffer Solution (PBS)	BasalMedia	Cat.B320KJ
Dulbecco's Modified Eagle Medium (DMEM)	BasalMedia	Cat.L110KJ
RPMI (Roswell Park Memorial Institute) 1640	BasalMedia	Cat.L210KJ
RIPA buffer	Beyotime	Cat.P0013B
Protease and phosphatase inhibitor cocktail	Beyotime	Cat.P1045
NP-40 lysis buffer	Beyotime	Cat. P0013F
Clodronate Liposomes	Ysasen	Cat.40337ES10
Protein A/G Magnetic Beads	Thermo	Cat.88803
Succinyl-Coenzyme A sodium	MCE	Cat. HY-137808
Evans Blue	MCE	Cat.HY-B1102,
Formamide	MCE	Cat.HY-Y0842
DAPI	Beyotime	Cat.1002C
Anti-DYKDDDDK Magnetic Agarose	Thermo	Cat.A36797
Anti-HA Magnetic Beads	Thermo	Cat.88836
TRIzol reagent	Invitrogen	Cat.15596026CN
Lipofectamine 3000	Thermo	Cat. L3000015
ECL UltraPlus Western HRP Substrate	Share-bio	Cat. SB-WB004
**Bacterial and virus strains**		
AAV6-Tie2-EGFP-shControl-WPREs	Genechem Co., Ltd	N/A
AAV6- Tie2-EGFP-shCcl7-WPREs	Genechem Co., Ltd	N/A
Trans5a Competent cell	TransGen Biotech	Cat.CD201
**Critical commercial assays**		
Foxp3 / Transcription Factor Staining Buffer Set	Thermo Fisher	Cat. 00-5523-00
HiScript IV All-in-One Ultra RT SuperMix for qPCR	Vazyme	Cat. R433-01
ChamQ Universal SYBR qPCR Master Mix	Vazyme	Cat. Q711-02
Mouse IL-6 ELISA Kit	MultiSciences	Cat.EK206
Mouse TNF-a ELISA Kit	MultiSciences	Cat.EK282
Mouse IL-1β ELISA Kit	MultiSciences	Cat.EK201B
Mouse CCL7/MCP-3 ELISA Kit	Solarbio	Cat. SEKM-0161

Human CCL7/MCP-3 ELISA Kit	Solarbio	Cat. SEKH-0514
TUNEL BrightRed Apoptosis Detection Kit	Vazyme	Cat. A113-01
Seahorse XF Mito Stress Assay Kit	Agilent	Cat. 103015-100
Seahorse XF Glycolytic Stress Test Kit	Agilent	Cat.103017-100
PKH26 Cell Membrane Staining Kit	Solarbio	Cat.D0030
DAB Detection Kit	GeneTech	Cat.GK600505
Masson's trichrome staining Kit	Solarbio	Cat. G1346
Chromatin Immunoprecipitation Kit	Sigma	Cat. 17-371
Dual-luciferase reporter assay	Promega	Cat.E1910
**Experimental models: Cell lines**		
HEK293T cells	This paper	N/A
**Experimental models: Organisms/strains**		
Mouse: C57BL/6	GemPharmatech Co., Ltd	N/A
Mouse: Knockout(Ccr1^{-KO})	GemPharmatech Co., Ltd	N/A
**Deposited data**		
Single-cell sequencing of mouse lung tissue	GEO	GSE207651
Whole blood transcriptome of septic patients	GEO	GSE54514
**Oligonucleotides**		
Primers for β-Actin;<br>Forward- CTAAGGCCAACCGT GAAAAG<br>Reverse- ACCAGAGGCATACAGGGACA	This paper	N/A
Primers for CCL7;<br>Forward-TCAAGAGCTACAGAAGGATCACC<br>Reverse-TGGAGTTGGGGTTTTCATGTCT	This paper	N/A
Primers for KAT2A;<br>Forward-AAGGCCAATGAAACCTGCAAG<br>Reverse-CTCACAGCTACGGCACAACTC	This paper	N/A
ChIP primers for GCK;<br>Forward-TTCAATTCCCAGCAACCACA,	This paper	N/A

Reverse-TCTATGCCTGGAAGCCTCAG		
ChIP primers for PFKL;<br>Forward- GGCCTGGGGAACCAGGGTTC,<br>Reverse- GTACCCGGTTTGTCCCGCCC	This paper	N/A
ChIP primers for HK1;<br>Forward- GGCACTTCCTGTAAGTCTTTTTGA,<br>Reverse- TTAATTCCCAGCAACCACACG	This paper	N/A
ChIP primers for HK2;<br>Forward-GTAGTGGCACATGCCACTC,<br>Reverse-ATTTATGAGTAGACGCCATCA	This paper	N/A
ChIP primers for LDHB;<br>Forward-CACATCTCTATCAAAGAGTCTCCTT,<br>Reverse-TGAGAATTTGCCTTTTGTTTTCTTT	This paper	N/A
ChIP primers for SLC2A1;<br>Forward-AGAGGCTATTGACTGTCCTACT,<br>Reverse-TTGGCATTGAACCAGCTCAC	This paper	N/A
Genotyping primers for CCR1;<br>Forward-CTCATGCAGCATAGGAGGCTT,<br>Reverse-ACATGGCATCACCAAAAATCCA	This paper	N/A
**Recombinant DNA**		
pCDH-puro-3xFlag-CCR1	This paper	N/A
pCDH-puro-3xFlag-STAT1^{-WT}	This paper	N/A
pCDH-puro-3xFlag-STAT1^{-K388R}	This paper	N/A
pCDH-puro-3xFlag-STAT1^{-K665R}	This paper	N/A
pCDH-puro-3xFlag-STAT1^{-K665E}	This paper	N/A
pCDH-puro-3xHA-KAT2A	This paper	N/A
HA-Ubiquitin	This paper	N/A
Myc-Ubiquitin	This paper	N/A
pGL3-basic-HK1^{-WT}	This paper	N/A

pGL3-basic-HK1^{-MUT}	This paper	N/A
pGL3-basic-LDHB^{-WT}	This paper	N/A
pGL3-basic-LDHB^{-MUT}	This paper	N/A
pGL3-basic-SCL2A1^{-WT}	This paper	N/A
pGL3-basic-SLC2A1^{-MUT}	This paper	N/A
**Software and algorithms**		
Image J	NIH	N/A
GraphPad Prism 8	GraphPad Software	N/A
Adobe Photoshop CC 2020	Adobe	N/A
CytExpert	Beckman	N/A
